# Energy and nitrogenous waste from glutamate/glutamine catabolism facilitates acute osmotic adjustment in non-neuroectodermal branchial cells

**DOI:** 10.1038/s41598-020-65913-1

**Published:** 2020-06-11

**Authors:** Pei-Chen Huang, Tzu-Yen Liu, Marian Y. Hu, Isabel Casties, Yung-Che Tseng

**Affiliations:** 1 Marine Research Station, Institute of Cellular and organismic Biology, Academia Sinica, I-Lan County, Taiwan (ROC); 2 Institute of Physiology, Christian-Albrechts University Kiel, Kiel, Germany; 3 Helmholtz Centre for Ocean Research Kiel (GEOMAR), Kiel, Germany

**Keywords:** Homeostasis, Animal physiology

## Abstract

Maintenance of homeostasis is one of the most important physiological responses for animals upon osmotic perturbations. Ionocytes of branchial epithelia are the major cell types responsible for active ion transport, which is mediated by energy-consuming ion pumps (e.g., Na^+^-K^+^-ATPase, NKA) and secondary active transporters. Consequently, in addition to osmolyte adjustments, sufficient and immediate energy replenishment is essenttableial for acclimation to osmotic changes. In this study, we propose that glutamate/glutamine catabolism and trans-epithelial transport of nitrogenous waste may aid euryhaline teleosts Japanese medaka (*Oryzias latipes*) during acclimation to osmotic changes. Glutamate family amino acid contents in gills were increased by hyperosmotic challenge along an acclimation period of 72 hours. This change in amino acids was accompanied by a stimulation of putative glutamate/glutamine transporters (Eaats, Sat) and synthesis enzymes (Gls, Glul) that participate in regulating glutamate/glutamine cycling in branchial epithelia during acclimation to hyperosmotic conditions. *In situ* hybridization of glutaminase and glutamine synthetase in combination with immunocytochemistry demonstrate a partial colocalization of *olgls1a* and *olgls2* but not *olglul* with Na^+^/K^+^-ATPase-rich ionocytes. Also for the glutamate and glutamine transporters colocalization with ionocytes was found for *oleaat1*, *oleaat3*, and *olslc38a4*, but not *oleaat2*. Morpholino knock-down of Sat decreased Na^+^ flux from the larval epithelium, demonstrating the importance of glutamate/glutamine transport in osmotic regulation. In addition to its role as an energy substrate, glutamate deamination produces NH_4_^+^, which may contribute to osmolyte production; genes encoding components of the urea production cycle, including carbamoyl phosphate synthetase (CPS) and ornithine transcarbamylase (OTC), were upregulated under hyperosmotic challenges. Based on these findings the present work demonstrates that the glutamate/glutamine cycle and subsequent transepithelial transport of nitrogenous waste in branchial epithelia represents an essential component for the maintenance of ionic homeostasis under a hyperosmotic challenge.

## Introduction

In a seawater (SW) environment, euryhaline teleosts passively lose water and gain salt. As a consequence, the fish must replenish water by drinking SW and actively excreting the majority of monovalent ions back to the environment, a process that occurs predominantly across gill epithelia^1,2^. Mitochondria rich (MR) cells in gill epithelia are majorly responsible for ATP-dependent active ion transport, which is mediated by various transporters and related enzymes. This homeostatic ion regulation against steep osmotic gradients has been demonstrated to be a highly energy consuming process^1,3–5^, and it has been demonstrated that gills respond to salinity fluctuations with increased metabolic demands^5,6^.

Various metabolic substrates are known to be utilized for energy generation in the gills of fish under salinity stress. On one hand, carbohydrate metabolism is a prime candidate to ensure timely delivery of an energy supply^4,5,7,8^. Recent studies have shown that acclimation to SW enhances glucose transport and utilization in gill epithelia of teleosts^5,8,9^, suggesting an increased carbohydrate requirement for osmotic adjustment. On the other hand, enhanced oxidation of amino acids (AAs) for ATP production has been well characterized in osmoregulatory organs (e.g., gill and intestine) during SW acclimation^10–12^. Furthermore, the role of non-essential AAs (NEAAs) in fish osmoregulation appears to be more prominent than that of essential AAs (EAAs)^5,13^. Among NEAAs, glutamate may be particularly important to serve as a potential substrate for fueling osmoregulation^14,15^. As such, glutamate content was reported to increase in gills of euryhaline teleosts following SW exposure^5,16^. Moreover, glutamate dehydrogenase (GDH) activity and glutamate content were also reported to be increased in isolated-gill epithelial cells in tilapia (*Oreochromis mossambicus*) following long-term (5 weeks) SW acclimation^17^. Interestingly, transaminase-mediated oxidation of branched-chain AAs (BCAAs) to glutamate led to the accumulation of glutamate in intestine, liver and serum of euryhaline fish after transfer to a high salinity environment^5^. Therefore, one cannot exclude the possibility that increased glutamate levels in gill tissue may originate from the serum. In fact, earlier studies have demonstrated that gills of rainbow trout (*Oncorhynchus mykiss*) are capable of taking up glutamate from the circulation^18^. In addition to its role as an energy substrate, glutamate acts as a non-toxic carrier of amine groups. Based on studies in mammals, the conversion of glutamate to NH_4_^+^ and α-ketoglutarate (α-KG) by GDH provides the NH_3_/NH_4_^+^ used to generate carbamoyl phosphate, an intermediate of urea synthesis^19,20^. Based on plasma composition, urea has been proposed to play a minor role as an osmolyte in teleosts^21,22^. Taking all of these previous findings into consideration we hypothesize that, glutamate may function as an important energy substrate during SW acclimation in euryhaline teleosts.

In the past five years, molecular physiological studies on experimental model teleosts, including tilapia and zebrafish, have provided new insights into ionocyte carbohydrate supply and transport during acclimation to environmental challenges. In tilapia, a novel type of gill cell, the glycogen-rich (GR) cell, was identified for its role in providing and storing energy to supply the Na^+^/ K^+^-ATPase-rich (NaR) cells in conditions of emergent energy demand^4,5,23^. The proposed model for metabolite exchange from teleost GR cells to gill epithelial ionocytes was further supported by recent physiological genomic and functional studies on zebrafish glucose transporters (drGLUTs) and manocarboxylate transporters (drMCTs)^2^. This energy exchange among gill non-neuroectodermal branchial cells is analogous to that between astrocytes and neurons in the brains of mammals^24^ and teleosts^25^. In brain, glycogen or glutamate is metabolized to lactate or glutamine and is shuttled from astrocytes to adjacent neurons, which have high energy requirements. Accordingly, it is possible that a glutamate-glutamine shuttle, similar to that found in neuroectodermal cells, may also exist in non-neuroectodermal branchial cells.

The export of glutamine from astrocytes and uptake by neurons are integral steps in the glutamate-glutamine cycle, a major pathway for glutamate replenishment in neurons. Thus, the participation of astrocytes in the glutamate-glutamine shuttle is critical for both metabolite transport and function of neurons. Mechanistically, the shuttle begins with the uptake of excess extra-synaptic glutamate and the production of glutamine via glutamine synthetase (Glul). This glutamine is then released from astroglia and taken up by neurons through the glutamine transporter, N-system A amino acid transporter (Sat), thereby replenishing the neuronal supply of glutamate^26–28^. Sat proteins are members of the solute carrier family 38 (SLC38) and were characterized as exhibiting high glutamine and alanine transport activities. The removal of glutamate from the synaptic cleft is mediated by high-affinity glutamate transporters of the excitatory amino acid transporter (Eaat) family^29,30^. Based on studies in mammals, Eaat1 (SLC1A3) and Eaat2 (SLC1A2) and Eaat3 (SLC1A1) account for more than 80% of all glutamate uptake activity in the nervous system^31,32^. In addition, it has also been reported that Eaat1 is functionally coupled to Na^+^-K^+^-ATPase (NKA)^30,32,33^, a primary transporter that drives neuronal ion gradients and is heavily involved in cellular homeostasis. Despite this detailed knowledge about glutamate cycling in neural tissue, the molecular and cellular processes that regulate metabolism and transport of glutamate/glutamine in non-neuroectodermal cell types, such as fish gill epithelial cells, are mostly unknown.

The present study aimed to test the hypothesis that glutamate/glutamine cycle may represent an energy provision that fuels osmo-regulatory processes in gill epithelium, similar to that found in mammalian brain tissue. In order to do so, we characterized key components of the glutamate-glutamine cycle in fish gills during acute exposure to hyperosmotic SW environment. Japanese medaka (*Oryzias latipes*), as euryhaline teleost, was selected as a model species due to its ability to adapt to acute 20‰ salinity brackish water (BW) challenges. We first utilized *in silico* cloning to identify the medaka genes for glutamate/glutamine transporters (Eaats and Sat), glutaminase (glutaminase, Gls) and glutamine synthetase (glutamate-ammonia ligase, Glul). We then further examined correlations between NH_3_/NH_4_^+^ secretion, content and NH_4_^+^-derived urea production in gills under hyperosmotic BW conditions in order to characterize these processes at an organismic level. Moreover, we determined the transcript levels of above mentioned genes in gills under FW and BW conditions. In addition, specific RNA probes were used to identify the cell types of the larval epithelium in which Eaats, Sat, Gls and Glul isoforms are predominantly expressed.

## Materials and Methods

### Experimental animals

Mature Japanese medaka (*Oryzias latipes*) were acquired from stocks of the Institute of Cellular and Organismic Biology, Academia Sinica, Taiwan. The fish were kept in circulating local freshwater (FW) at 27–28 °C with a 12:12 h light-dark photoperiod. Fertilized egg clusters were collected from the belly of a female. At the 7 day post fertilization (dpf), larvae were used for *in situ* hybridization and immunostaining experiments. Experimental protocols and all methods were approved and performed in accordance with the relevant guidelines and regulations by the Academia Sinica Institutional Animal Care and Utilization Committee (approval no. RFIZOOHP220782).

### Hyperosmotic brackish water transfer experiments

Brackish water with 20‰ salinity was prepared by adding artificial sea salt (Taikong, Taipei, Taiwan) to aerated FW. Before the salinity transfer experiments, FW medaka were starved for 24 h. After starvation, medaka were transferred from FW to FW (control group) or 20‰ brackish water (BW) (treatment group), and were sampled at 0, 6, 24 and 72 h after transfer for metabolic measurements. Fish were not fed during the experimental period. Before each sampling, fresh wet mass (WM) of the adult fish was recorded, and fish were subsequently anesthetized with MS222 and sacrificed by a cut through the spine. The gill tissues were taken, weighed and prepared for examination of gene expressions, FAA contents and histological features.

### Oxygen consumption and NH_4_^+^ excretion

Oxygen consumption was determined before the start of the experiment (0 h) and at further sampling time points of 6, 24 and 72 h, and followed procedures modified from^34,35^. Medaka were gently transferred to a 0.15 L glass respiration chamber, containing 0.2 μm filtered FW or 20‰ BW. Respiration chambers were sealed without any air inside, and submerged in a water bath at 27 °C. Oxygen concentration inside the chamber was recorded using a fiber optic oxygen sensor (PreSens sensor spots, type PSt3) in the chamber lid that was connected to an OXY-4 mini multichannel fiber optic oxygen transmitter (PreSens, Regensburg, Germany). The sensors were calibrated according to the manufacturer’s instructions. Preliminary experiments demonstrated that the swimming movements of the experimental animal could sufficiently mix the water inside the respiration chamber, resulting in a measured linear decrease of oxygen concentrations inside the chamber. When the oxygen concentration reached 75% of the air saturation level, animals were removed from the respiration chamber. Additionally, a separate glass chamber was incubated without an experimental animal to determine background readings of filtered FW or 20‰ BW and check for potential bacteria contamination. Oxygen consumption rates were calculated based on the linear decrease in oxygen concentration during the interval, beginning from 5 min after the start of the experiment to the end of the measurement period. The first 5 min were discarded to ensure that the animal was sufficiently acclimated to the new environment and prevent artifacts due to handling stress. After oxygen consumption was measured, the wet mass of individuals was recorded and oxygen consumption rates were calculated as μmole O_2_ h^−1^g_*WM*_^−1^.

Ammonium excretion by medaka was measured using a method previously described by Holmes *et al*.^36^. For the determination of ammonia excretion rates, water samples were collected from the FW and 20‰ BW before fish were transferred and after each sampling time point. Water samples (25 μL) were mixed in a 96-well black microplate with 100 μL of NH_4_^+^ assay reagent, containing orthophthaldialdehyde. The mixture was incubated at room temperature for 150 min, after which the microplate was read on a Spectra Max M5 microplate reader (Molecular Devices, CA, USA), at excitation/emission wavelengths of 360/420 nm. The atomic ratio of oxygen uptake and excreted nitrogen was calculated from respiration and ammonium excretion rates as:

$${\rm{O}}:\,{\rm{N}}=2\,{\rm{M}}{{\rm{O}}}_{2}{({\rm{N}}{{{\rm{H}}}_{4}}^{+}{\rm{e}}{\rm{x}}{\rm{c}}{\rm{r}}{\rm{e}}{\rm{t}}{\rm{i}}{\rm{o}}{\rm{n}})}^{-1}$$

### Free AA and ammonia content

Total AAs were extracted from gill samples using 2.5 mL ethanol with 12.5 nmol norvaline. After homogenization and centrifugation at 4,300 × *g* for 10 min, 2 mL of supernatant was transferred to a new tube, and dried in a vacuum concentrator (Concentrator 5301). The dried samples were reconstituted in 100 μL of 8 mM HCl and extruded through a 0.2-µm syringe filter (Millipore Syringe Filters, Millipore Millex, France), after which samples were derivatized using a commercial kit (AccQ Tag Ultra Reagent Kit, 186003836, Waters, Milford, MA, USA). The derivatized samples were measured using ultra-performance liquid chromatography (UPLC) (ACQUITY UPLC H-Class System, Waters). The system was equipped with a BEH C18 column and a TUV detector. Individual AAs and derived ammonia were quantified from the chromatogram by comparison of retention times and peak areas to known standards (WAT088122, Waters).

### Urea content

Urea content in medaka gills was measured with a commercial colorimetric urea assay kit (MAK006, Sigma-Aldrich, St. Louis, MO, USA). Gill tissue was rapidly homogenized in 100 μL of cold urea assay buffer, and centrifuged at 13,000 × *g* for 10 min at 4 °C to collect the supernatant. The samples were mixed with assay reagent in a clear 96-well microplate and then incubated at 37 °C for 60 min. Absorbance was measured at 570 nm with a microplate reader (Spectrophotometer, Thermo scientific, MultiSkan GO, NH, USA).

### Purification of total RNA

Total RNA was extracted from gills, brain, liver, intestine and muscle of medaka. Tissues were homogenized in TRIzol reagent (Invitrogen, Carlsbad, CA, USA) and treated with DNase I (Promega, Madison, WI, USA) to remove genomic DNA contamination. Total RNA was purified following the manufacturer’s protocol. The amount and quality of mRNA was determined at 260/280 nm absorbance spectrophotometry (ND-2000, NanoDrop Technol, Wilmington, DE, USA). The RNA integrity was further double check with Agilent 2100 bioanalyzer (Agilent Technologies, Santa Clara, CA) as shown in Supplemental Fig. S1. All the stringent-examined RNA samples were stored at −20 °C.

### *In-silico* cloning and reverse-transcription polymerase chain reaction (RT-PCR) analysis

*In-silico* predicted of candidate homologues in medaka were carefully obtained from the Japanese medaka HdrR genome database (Ensembl Genome Browser system; ver. ASM223467v1). To verify the identified candidates belong to the respective protein orthologue, the deduced amino-acid sequences of medaka genes were aligned with ClustalX together with those known protein sequences available from the genome database (Ensembl Genome Browser system) or NCBI database. Furthermore, specific primers (as listed in Table 1) were designed for cloning by the reverse-transcriptase polymerase chain reaction (RT-PCR).

For cDNA synthesis, 5 μg of mRNA was reverse transcribed in a final volume of 20 μL, containing 0.5 mM dNTPs, 2.5 mM oligo(dT)_20_, 250 ng of random primers, 5 mM dithiothreitol, 40 units of RNase inhibitor, and 200 units of SuperScript III RT (Invitrogen, Carlsbad, CA, USA) for 1 h at 50 °C, followed by incubation at 70 °C for 15 min. The amount and quality of cDNA were determined at 260 and 280 nm by the Qubit dsDNA HS Assay Kit on the Qubit Fluorometer (Life Technologies, CA, USA). For PCR amplification, 1 μL of cDNA was used as a template in a 25 μL final reaction volume, containing 0.25 mM dNTPs, 2.5 units of Gen-Taq polymerase (GeneMark, Taipei, Taiwan), and 0.2 μM of each primer (Table 1

**Table 1:** Primers used for RT-PCR cloning and qPCR.

**Protein name**		**Abbreviation**	**Gene name**	**Gene loci**	**Primer sequence (5′->3′)**		**Amplicon** **size (bp)**	**Primer efficiency (%)**	**Accession number**
**Glutamate/ Glutamine cycle**	Glutamate transporter	Eaat3	*olslc1a1*	Primary_assembly 18: 39.45 Mb	Cloning		555		ENSORLT00000015091
					F	TCAGAGAGTTTGCACCGCTT			
					R	GGCCGAAAGCAACACAGAAG			
					qPCR		284	97~98	
					F	TGTTGGGCTTGATCGTCTTC			
					R	GAAGTAGATGAGAGGAAGGCAG			
		Eaat2	*olslc1a2a*	Primary_assembly 6: 22.95 Mb	Cloning		819		ENSORLT00000015112
					F	CCTGAAAACCTGGTGCAAGC			
					R	AGCGGTCAAGATCAACAGCA			
					qPCR		191	95~98	
					F	GTGGAGGTGAGAATGCATGAGAGT			
					R	ACCATGACGATGTCTGGGTGGATA			
		Eaat2	*olslc1a2b*	Primary_assembly 3: 8.15 Mb	Cloning		762		ENSORLT00000001177
					F	CGAAATCCAGTGGCCGTTTG			
					R	GCTTGTCGATGCCCAAGTTC			
					qPCR		122	91~92	
					F	AGCAGGTCAGGATGGATGACTTTG			
					R	CATGGTGACCTTGCAGTCGTCTAT			
		Eaat1	*olslc1a3*	Primary_assembly 9: 11.03 Mb	Cloning		617		ENSORLT00000015112
					F	ATGCCCTGGGCTTGGTAATG			
					R	TATTCCAGCTGCTCCGATGC			
					qPCR		144	93~94	
					F	GTCTTCAGCAAATCAGTGCCAGGA			
					R	TGCCTATGATTACAGCAGCCACAG			
	Glutamine transporter	Sat	*olslc38a4*	Primary_assembly 23: 4.09 Mb	Cloning		650		ENSORLT00000012289
					F	GGGCCTTCATGGGCTTAGAG			
					R	CATGTTGATGGAGGAGCGGA			
					qPCR		191	87~91	
					F	AGTTTGACACCTTGCTGCTGTTGG			
					R	GCAGATGACCAGCGTGTTGTTGAA			
			*olslc38a5*	Primary_assembly 7: 21.08 Mb	Cloning		616		ENSORLT00000017690
					F	CGGAGCTGCCTCTGGTTATT			
					R	ACGGTCAGAATGACGGCTAC			
					qPCR		92	96~97	
					F	TCCTCTCATCTCCACATCTCC			
					R	TGTATGCCGTCTGTGAGTTG			
	Glutamine synthetase	Glul	*olglul*	Primary_assembly 17: 25.08 Mb	Cloning		691		ENSORLT00000020876
					F	AGCAACCTTCGGATCACCTG			
					R	GGCGGTCTTCAAAGTAGCCT			
					qPCR		131	99~100	
					F	TTGGACCTTGTGAGGGAATCGACA			
					R	TTGTGTGGCACCATTCCAGTTTCC			
	Glutaminase	Gls	*olgls1a*	Primary_assembly 2: 3.97 Mb	Cloning		979		ENSORLT00000000181
					F	CGAACTCTACGAGAACGCCA			
					R	CAGCAGGTTGATGACGGACT			
					qPCR		173	95~96	
					F	TAAAGTCAACCCGGTTCCCAAGGA			
					R	ACAGCATCCCGTCCAGCTTATTCT			
			*olgls1b*	Primary_assembly 21: 16.57 Mb	Cloning		960		ENSORLT00000018999
					F	AGCCACGCATTTCTCACTCA			
					R	CAGCGTTCACCATTGGGTTG			
					qPCR		117	91~95	
					F	AACACCAATGGATGAGGCAATGCT			
					R	TTCTCCGTCCTCTCTTGTCCATCA			
			*olgls2*	Primary_assembly 7: 19.29 Mb	Cloning		956		ENSORLT00000015645
					F	ACAGTTCACCCACCAGATCG			
					R	TCCTGGGATCGTGCTTCTTG			
					qPCR		110	95~98	
					F	GACGCCGTTGTTTCTATCCTGCAA			
					R	-AGAGAGCTCTTCATGCTGTCTAGG			
**Urea cycle**	Carbamoyl phosphate synthetase	Cps I	*olcps1*	Primary_assembly 21: 18.51 Mb	Cloning		910		ENSORLT00000020045
					F	CTGAGAAAACCACCGCTTGC			
					R	CCTCCCACTGACACAATGCT			
					qPCR		105	94~97	
					F	ATGAGTGTGACCGCCTCTACTT			
					R	TCTGACCTCCCACTGACACAAT			
		Cps II	*olcps2*	Primary_assembly 24: 1.08 Mb	Cloning		782		ENSORLT00000010929
					F	TGAGGAACACGGCCATCAAA			
					R	CAGGTAGTTGGTGTAGGCGG			
					qPCR		120	97~98	
					F	ACTACCTGTACCTGACCTACC			
					R	CACACCAGTCGAACTCCAC			
	Ornithine transcarbamoylase	Otc	*olotc*	Primary_assembly 21: 3.90 Mb	Cloning		452		ENSORLT00000011358
					F	GCTCGTCTTGGATCGGTGAG			
					R	TGCAGAGTGAGAAAGTCCGC			
					qPCR		170	91~94	
					F	CCTTGTTTCCTCACCTCACAAGAC			
					R	GACAGACCGTTGATGATGGGAATG			
**Ammonia transport**	Rhesus protein	Rhbg	*olrhbg*		qPCR		197	95~97	NM_001105091.1
					F	CGCTGTGACTCTGGGCAT			
					R	GCTTGTCGGACTCCTCTG			
		Rhcg	*olrhcg1*	Primary_assembly 6: 22.09 Mb	Cloning		547		ENSORLT00000014707
					F	ATCACTGAAGTGTGTCGGGG			
					R	TGGCGAGACCATAGTAGGCT			
					qPCR		117	90~94	
					F	CTTGGGAGATGATGGGAAGATAAG			
					R	GCTGGACTGGACTGACTTTAC			
**Reference gene**	Ribosomal protein L7	Rpl7	*olrpl7*		qPCR		105	97~99	NM_001104870
					F	GAGATCCGCCTGGCTCGTA			
					R	GGGCTGACTCCGTTGATACCT			

F, forward primer; R, reverse primer.

). For each reaction, PCR was performed for forty cycles. PCR products were then subcloned into a pGEM-T Easy vector (Promega, Madison, WI, USA), and the nucleotide sequences were determined with an ABI 3730XL sequencer (Applied Biosystems, Warrington, UK). Sequence analysis, alignment, and confirmation were carefully conducted with both the BLASTx program (NCBI) and the BLAST/BLAT search program (Ensembl).

### Real-time quantitative PCR (qPCR) analysis

Total RNA was extracted and reverse-transcribed from gill tissue as described. The mRNA expressions of target genes (as listed in Table 1) was measured by qPCR using the Roche LightCycler® 480 System (Roche Applied Science, Mannheim, Germany). PCRs contained 5 ng of cDNA, 50 nM of each primer, and the LightCycler® 480 SYBR Green I Master (Roche) in a final volume of 10 μL. All qPCR reactions were performed as follows: 1 cycle of 50 °C for 2 min and 95 °C for 10 min, followed by 40 cycles of 95 °C for 15 s and 60 °C for 1 min (the standard annealing temperature of all primers). PCR products were subjected to a melting-curve analysis, and representative samples were electrophoresed to verify that only a single product was present (as shown in Supplemental Fig. S2). Control reactions were conducted with sterile water to replace cDNA sample as non-template control (NTC). The standard curve of each gene was confirmed to be in a linear range with ribosomal protein L7 (*olrpl7*) as a reference gene. The expression of this reference gene has been demonstrated to be stable among ontogenetic stages and during acid-base perturbation treatment in Japanese medaka^37,38^.

### RNA probe synthesis

Fragments of glutamate/glutamine cycle-related gene isoforms were obtained by PCR and inserted into the pGEM-T easy vector (Promega). The T7 and SP6 primers were used to amplified the inserted fragments by PCR. The DIG-labeled RNA probes containing sense and anti-sense probes (Supplemental Table S1) were synthesized by *in vitro* transcription with T7 and SP6 RNA polymerase (Roche, Penzberg, Germany). The quality and concentrations of digoxigenin (Dig)-labeled RNA probes were examined using RNA gels and a dot blot assay.

### Whole mount *in situ* hybridization and immunofluorescence staining

Medaka larvae (7 dpf) were anesthetized with MS222 and then fixed with 4% paraformaldehyde in a phosphate-buffered saline (PBS) solution at 4 °C overnight. Afterward, samples were washed with diethylpyrocarbonate (DEPC)-PBST (PBS with 0.1% Tween-20) several times for 10 min each wash. After a brief rinse with PBST, larvae were incubated with hybridization buffer (HyB: 50% formamide, 5× saline-sodium citrate (SSC), and 0.1% Tween 20) at 65 °C for 5 min and with HyB containing 500 μg/ml yeast tRNA at 65 °C for 4 h before hybridization. After overnight hybridization with 100 ng/ml DIG-labeled antisense (or sense) RNA probes, larvae were serially washed with 50% formamide-2× SSC (at 65 °C for 20 min), 2× SSC (at 65 °C for 10 min), 0.2× SSC (at 65 °C for 30 min, twice), and PBST at room temperature for 10 min. RNA fluorescence staining was conducted with the commercial kit, TSA Plus Fluorescence Systems (Perkin Elmer, Boston, MA, USA). Fluorescence signals detected by DIG-labeled RNA probes were enhanced through fluorescein-tyramide signal amplification. It was an enzyme-mediated detection method to generate high-density labeling of a target nucleic acid. Images were acquired by Leica TCS-SP5 confocal laser scanning microscope (Leica Lasertechnik, Heidelberg, Germany).

For double-labeling with candidate genes mRNA and Na^+^-K^+^-ATPase (NKA), the samples were first *in situ* hybridized with specific RNA probe and subsequently subjected to immunocytochemical treatments. After washed with PBS, the *in situ* hybridized samples were incubated in 1% blocking solution for 1 h. Samples were then incubated overnight at 4 °C with α5-monoclonal antibody (anti-avian NKA α subunit diluted 1:200 with PBST; Developmental Studies Hybridoma Bank, University of Iowa, Ames, IA). Moreover, samples were incubated in goat anti-mouse IgG conjugated with Alexa Fluor 568 (Molecular Probes, Carlsbad, CA, USA, diluted 1:200 with PBST) for 2 h at room temperature. After washing with PBS (3 × 5 min), images were obtained by Olympus FV3000 confocal laser scanning microscope (Olympus Corporation, Tokyo, Japan).

### Knockdown of protein translation with antisense morpholino oligonucleotides

Morpholino-modified antisense oligonucleotides (MOs) were purchased from Gene Tools (Philomath, OR, USA). The sequence of the MO targeting *olslc38a4* (XM_011490891.2) was 5′-CTGGAAGCGTCAACATGCCGAGATT-3′; this MO was prepared with 1× Danieau solution. Standard control MO (provided by Gene Tools) with a non-specific sequence (5′-CCTCTTACCTCAGTTACAATTTATA-3′) was injected in parallel as a ‘sham’ control. Medaka embryos at the one-cell stage were injected with a 0.1% phenol red (colored indicator)-containing MO solution, using an IM-300 microinjection system (Narishige Scientific Instrument Laboratory, Tokyo, Japan). Various dosages (0.5, 1, 2 and 4 ng per embryo) of MO were assessed; the 1 ng-injected group exhibited a regular phenotype with slightly malformation rate (<29%) compared with 2 ng- and 4 ng-injected groups (malformation rate: 84% for 2 ng injection and 73% for 4 ng injection). Therefore, 1 ng per embryo was used in all subsequent experiments.

### Scanning ion-selective electrode technique (SIET)

SIET was used to measure Na^+^ flux activity at the epithelium surface of medaka larva. Glass capillary tubes (no. TW 150 – 4; World Precision Instruments, Sarasota, FL) were pulled on a Sutter P-97 Flaming Brown pipette puller (Sutter Instruments, San Rafael, CA) into micropipettes with tip diameters of 3–4 μm. The micropipettes were then baked at 120 °C overnight and coated by incubation with dimethyl chlorosilane (Sigma-Aldrich) for 30 min. The micropipettes were backfilled with a 1-cm column of electrolytes and frontloaded with a 20–30 μm column of liquid ion-exchange cocktail (Sigma-Aldrich) to create an ion-selective microelectrode (probe). The ionophore cocktail (and electrolytes) was Na^+^ ionophore II cocktail A (100 mM NaCl). To calibrate the ion-selective probe, the Nernstian response of each microelectrode was evaluated by placing it in a series of standard solutions (0.1, 1, and 10 mM NaCl dissolved in distilled water). By plotting the voltage output of the probe against log [Na^+^] value, linear regression yielded a Nernstian slope of 56.7 ± 0.5 (N = 10). In preliminary tests, the selectivity of the Fluka Na^+^ ionophore II cocktail A was 10–16 times more selective to Na^+^ than to NH_4_^+^ (measured in 1–10 mM Na^+^ solution).

### Measurement of Na^+^ gradients from epithelium

The SIET measurement of Na^+^ gradients was measured following the method described in^39,40^, using Na^+^ selective microelectrodes. SIET was performed at room temperature (26–28 °C) in a small plastic recording chamber filled with 2 ml of normal recording medium that contained 0.5 mM NaCl, 0.2 mM CaSO_4_, 0.2 mM MgSO_4_, 300 μM MOPS buffer, and 0.3 mg l^−1^ ethyl 3-aminobenzoate methanesulfonate (Tricaine, Sigma-Aldrich). Before measurement, an anesthetized larva was positioned in the center of the chamber with its lateral side contacting the base of the chamber. After a 3 min wait for signal stabilization, the ion-selective probe was moved to the target position (10–20 μm away from the yolk sac epithelium) to record the ionic activities for 10 s; then the probe was immediately moved away (1 cm) to record the background for another 10 s. To calculate ionic gradients, the background concentration was subtracted from the concentration at the target position. In this study, Δ[Na^**+**^] represents the measured Na^**+**^ gradients between the target position (at the surface of larval epithelium) and background. The noise of the system was usually less than 10 μV and was neglected when calculating ionic gradients, as the recorded voltage difference with larvae was usually 1–10 mV.

### Statistical analysis

GraphPad Prism 7.00 (GraphPad, San Diego, CA, USA) was used for statistical analyses. Values are presented as mean ± SD. Student’s t-test was used to analyze difference of oxygen consumption rate, ammonia extraction rate, O:N ratio, and free AAs contents in fish between FW control and 20‰ BW treatment. Two-way analysis of variance (ANOVA) and Tukey’s HSD test was used to determine the effect of salinity variance and time course treatment on target genes expressions along the experiments. One-way ANOVA followed by Tukey’s pairwise comparison was used to determine the effect of morpholino knockdown on Na^+^ gradients from the yolk sac epithelium. Differences were considered to be significant at *p* < 0.05.

## Results

### Effects of salinity challenge on metabolic responses of intact fish

**Figure 1:**
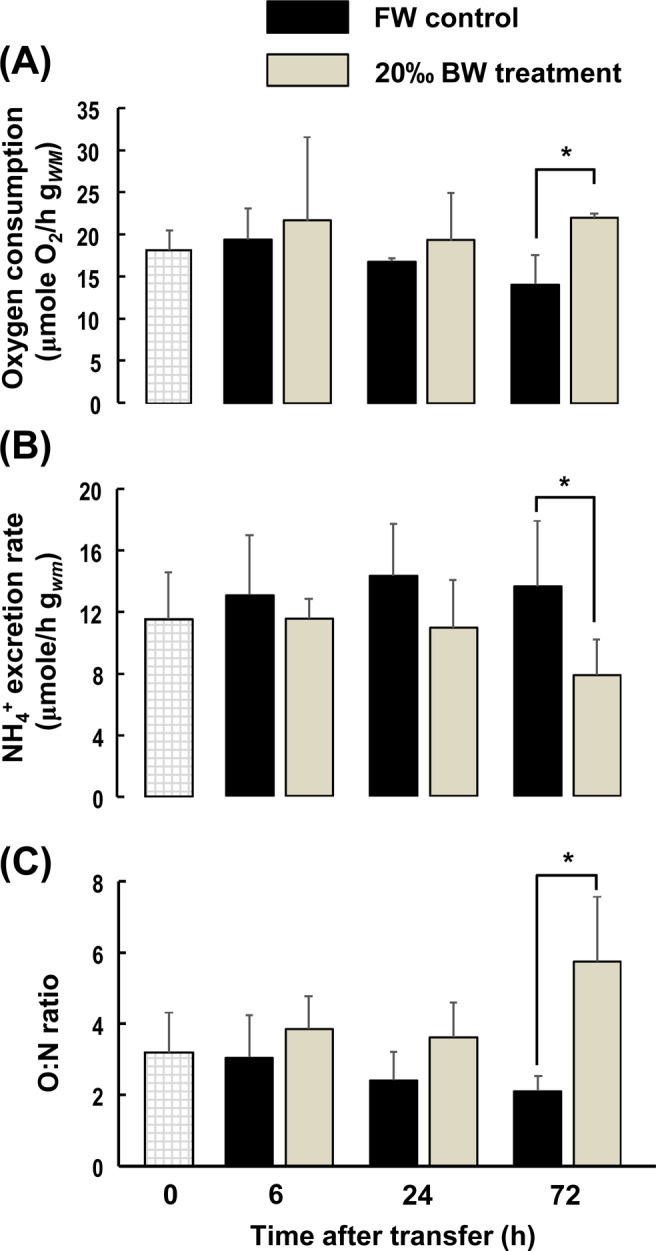
Effects of salinity on metabolic rates and ammonia excretion. Routine metabolic rates were determined by oxygen consumption 6, 24 and 72 h after transferring fish to FW or 20‰ BW environments. (**A**) Ammonium (NH_4_^+^) excretion rates were determined by NH_4_^+^ concentrations in bath water 6, 24 and 72 h after transferring fish to FW or 20‰ BW. (**B**) O:N ratios were calculated from oxygen consumption and NH_4_^+^ excretion and are shown along the same time course of exposure to FW or 20‰ BW. (**C**) Data are presented as mean ± SD (n ≥ 6). An asterisk (*) indicates significant difference, *p* < 0.05, between 20‰ BW and FW groups at the same time point.

The metabolic rates of medaka kept under control FW conditions were 18.13 ± 2.30 μmol O_2_ h^−1^ g_*WM*_^−1^ (Fig. 1A). During short-term (6 h and 24 h) exposures to hyperosmotic BW conditions, no significant differences in respiration rates were observed. However, after exposure to 20‰ BW for 72 h, the metabolic rate was 22.07 ± 0.55 μmol O_2_ h^−1^ g_*WM*_^−1^, which corresponds to 56.5% significant increase compared to FW controls at the same time point (14.11 ± 3.49 μmol O_2_ h^−1^ g_*WM*_^−1^) (Fig. 1A). After exposure to 20‰ BW conditions for 72 h, whole animal NH_4_^+^ excretion rates were significantly decreased by about 44% (Fig. 1B). Consequently, a major change in the O:N ratio was observed in animals treated with 20‰ BW for 72 h compared with FW controls (Fig. 1C).

### Accumulation of glutamate-related AAs in gills after BW transfer

**Figure 2:**
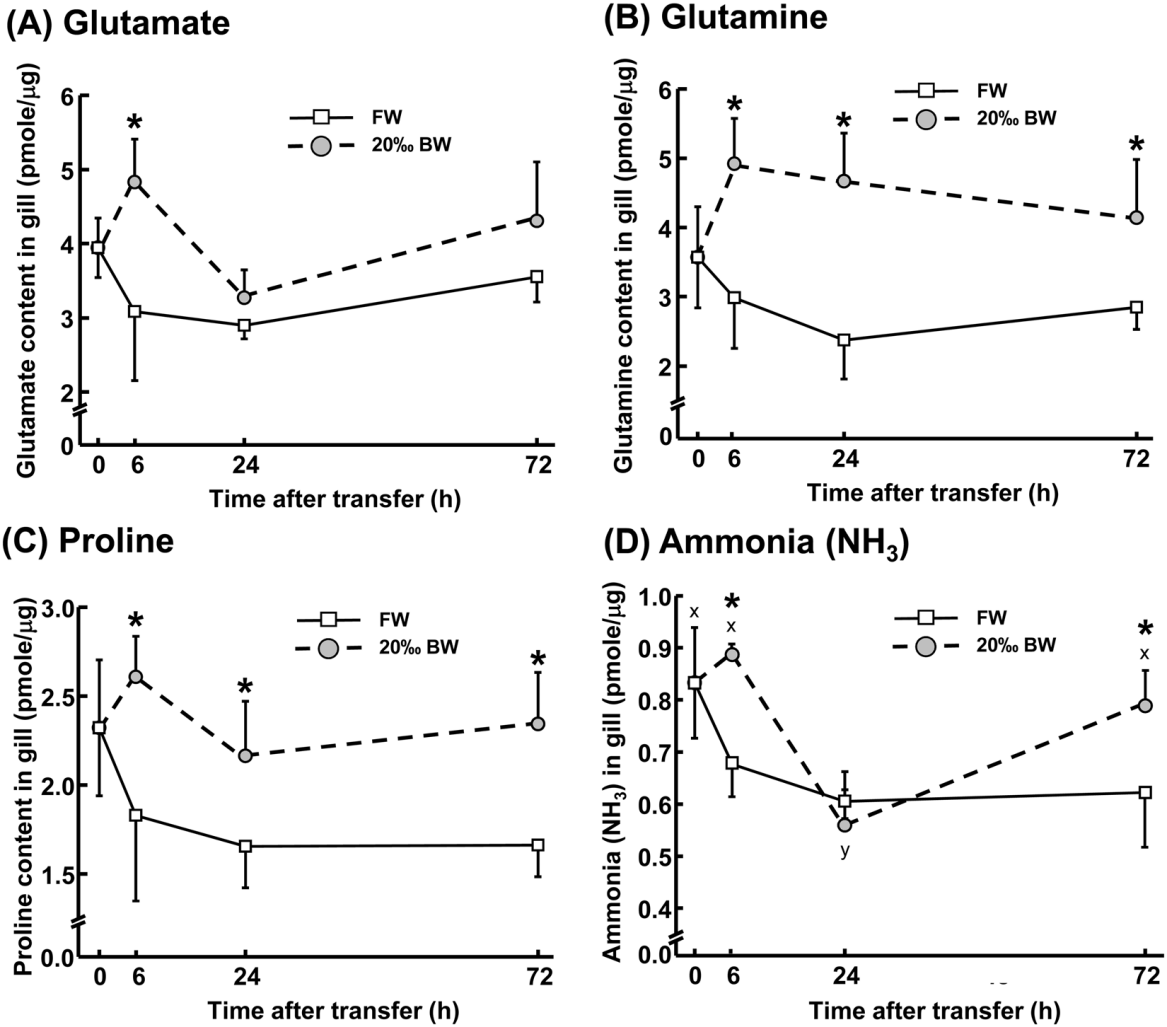
Amino acid content (pmole/μg) in the gills of adult Japanese medaka *Oryzias latipes* during FW and 20‰ BW exposures. Contents of glutamate (**A**), glutamine (**B**), proline (**C**) and derived ammonia (NH_3_) in the gills of adult medaka were measured by ultra-performance liquid chromatography (UPLC). Data are expressed as mean ± SD (n = 4–6). An asterisk (*) indicates significant difference, *p* < 0.05, between FW and 20‰ BW groups at the same time point. Different letters indicate significant differences between time points in each treatment group (Two-way ANOVA and Tukey’s HSD test).

The effects of BW hyperosmotic conditions on glutamate/glutamine shuttle-related AA levels were examined in gill tissue. Glutamate content in gills was significantly increased by ~55% in animals exposed to 20‰ BW for 6 h compared to animals kept under FW conditions (Fig. 2A). In addition to the observed increases in glutamate, glutamine contents in gill tissues were increased by ~64% and ~114% after transfer to 20‰ BW condition for 6 h and 24 h, respectively (Fig. 2B). Similarly, the concentration of proline, one of the substrates for glutamate production, was also found to increase after transfer to 20‰ BW condition. Proline was increased by ~63% upon acute BW exposure (6 h) compared to the FW control group (Fig. 2C). The level of nitrogenous ammonia (NH_3_/NH_4_^+^) waste in gills, which may be derived from glutamine deamination, was also found to be increased by 31% and 25% after transfer to 20‰ BW for 6 h and 72 h, respectively (Fig. 2D). In addition, other carbon cycle-related AAs were as well estimated in this study. After transfer to 20‰ BW condition, contents of AAs in gills that involve in converting to pyruvate and acetyl CoA were found to be apparently responsive to hyperosmic challenges (Supplemental Fig. S3).

### Ammonia transport and urea production in gills

**Figure 3:**
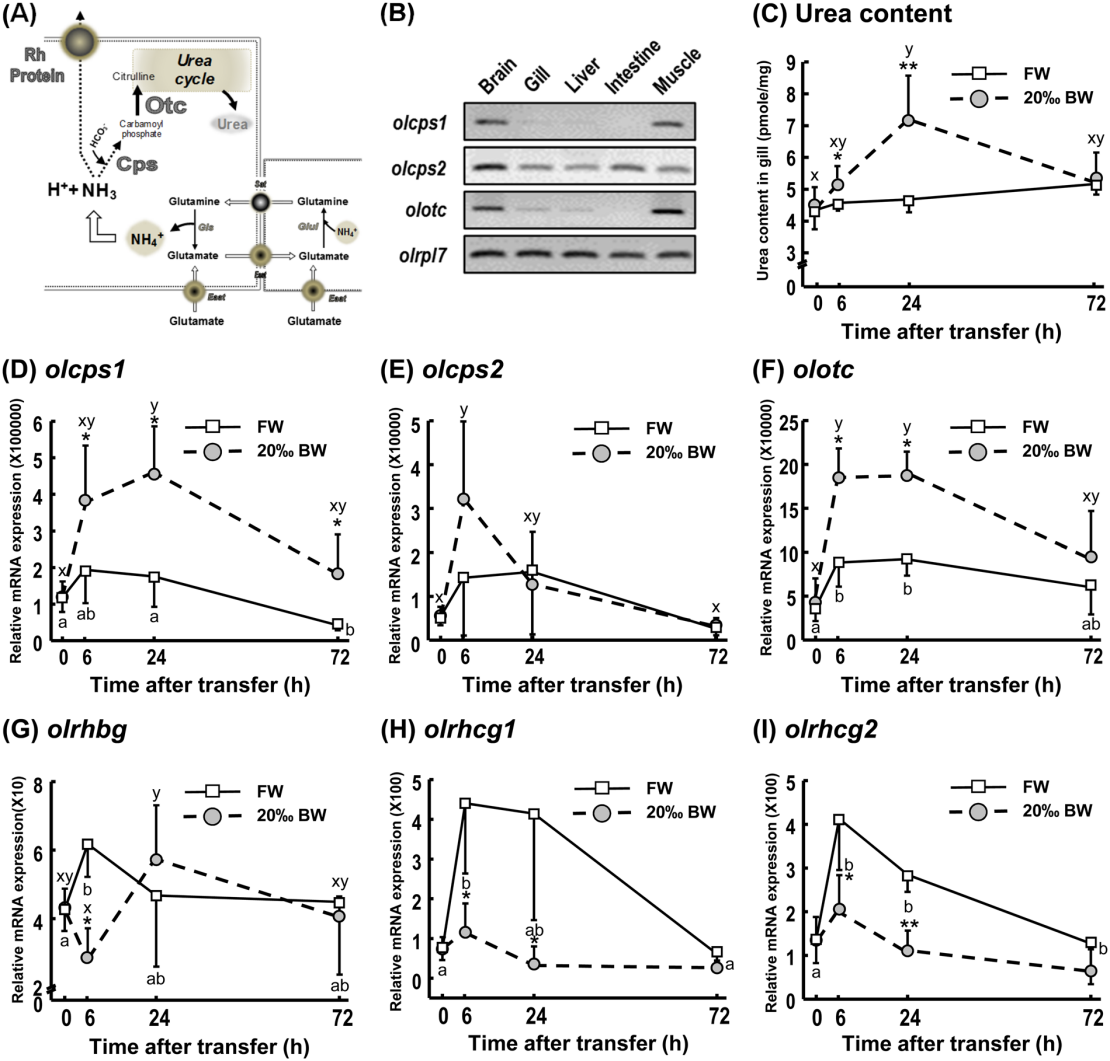
Gene expression of urea content, urea cycle-related enzymes and Rh ammonia transport transporters in gills during FW and 20‰ BW exposures. A schematic model of nitrogenous waste NH_3_/NH_4_^+^ transport and metabolism is shown (**A**). Cropped agarose gels (original images as shown in Supplemental Fig. S6) show semi-quantitative PCR (with 40-cycle amplification) of urea cycle-related enzymes, carbamoyl phosphate synthetase (*olcps1* and *olcps2*), ornithine transcarbamylase (*olotc*) and the reference gene ribosomal protein L7 (*olrpl7*) in brain, gill, liver, intestine and muscle of adult medaka (**B**). Urea content in gill (ng/mg) was estimated during exposure to FW or 20‰ BW (F) (n ≥ 10) (**C**). Quantitative PCR (qPCR) of carbamoyl phosphate synthetase (*olcps1* (**D**), *olcps2* (**E**)), ornithine transcarbamylase (*olotc* (**F**)) and Rh ammonia transport transporters (*olrhbg* (**G**), *olrhcg1* (**H**), *olrhcg2* (**I**)), in gills of adult medaka transferred to FW or 20‰ BW. *olrpl7* was used as the reference gene. Data are expressed as mean ± SD (n = 4–6). Asterisk indicates significant difference (*p < 0.05, **p < 0.01) at same time point between FW and 20‰ BW groups. Different letters indicate significant differences between time points in each treatment group (Two-way ANOVA and Tukey’s HSD test). Overlapping letters indicate that differences are not significant between time points in the treatment group.

The oxidative deamination of FAAs produces nitrogenous waste ammonium (NH_4_^+^), which is secreted to the extracellular environment via ammonia transporters, such as Rhesus (Rh) proteins, or detoxified by the urea cycle (Fig. 3A). The urea content in gill tissue was significantly increased at 6 h (19%) and 24 h (26%) after transfer to 20‰ BW, compared with FW controls (Fig. 3C). Transcript levels of rate-limiting mitochondrial enzymes carbamoylphosphate synthetases (CPS) in gills for urea synthesis (Supplemental Fig. S4A), *olcps1*, was upregulated in the 20‰ BW treatment group (Fig. 3D). Beside, 20‰ BW did not apparently induce *olcps2* expression at any of the treatment times (Fig. 3E). In addition, ornithine carbamoyltransferase (*olotc*) in gills (Supplemental Fig. S4A), which is also a rate-limiting enzyme of the urea cycle in mitochondria, was observed to be significantly upregulated in gills throughout the 20‰ BW treatment (Fig. 3F). However, Rh proteins were all downregulated in gill tissue upon salinity challenge (Fig. 3G–I), which is correspondence with previous studies in medaka as well^41^.

### Glutamate and glutamine synthesis in fish gill epithelium during salinity challenge

**Figure 4:**
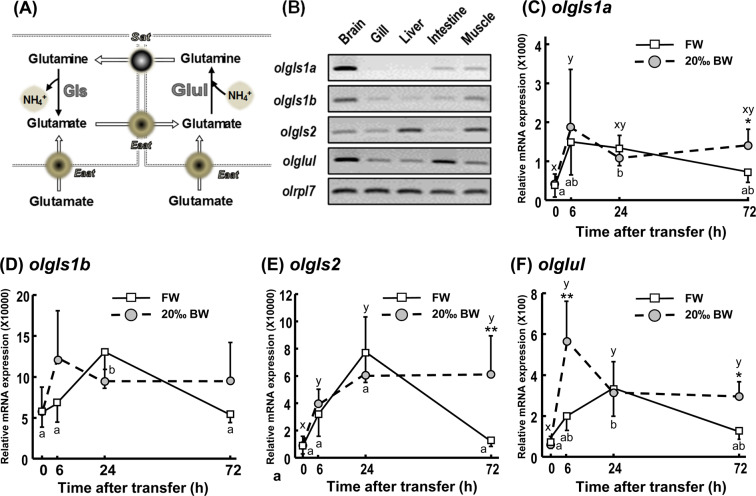
Glutaminase (*olgls*) and glutamine synthetase (*olglul*) gene expression in gills during FW and 20‰ BW exposure. A schematic of the glutamate-glutamine cycle and putative enzymes is shown. (**A**) Cropped agarose gels (original images as shown in Supplemental Fig. S6) show semi-quantitative PCR (with 40-cycle amplification) of glutaminase (*olgls1a*, *olgls1b* and *olgls2*), glutamine synthetase (*olglul*) and the reference gene ribosomal protein L7 (*olrpl7*) in brain, gill, liver, intestine and muscle of adult medaka. (**B**) Quantitative PCR (qPCR) analysis of glutaminase and glutamine synthetase genes, including *olgls1a* (**C**), *olgls1b* (**D**), *olgls2* (**E**), and *olglul* (**F**), in gills of adult medaka transferred to FW or 20‰ BW. *olrpl7* was used as the reference gene. Data are expressed as mean ± SD (n = 4–6). Asterisk indicates significant difference (*p < 0.05, **p < 0.01) at same time point between FW and 20‰ BW groups. Different letters indicate significant differences between time points in each treatment group (Two-way ANOVA and Tukey’s HSD test). Overlapping letters indicate that differences are not significant between time points in the treatment group.

GLS enzymes are responsible for catalyzing glutamate synthesis (Fig. 4A), and three Gls candidates were identified in medaka gills (Fig. 4B; Supplemental Fig. S4B). Transcript levels of these genes were differentially regulated during exposure to 20‰ BW conditions along the 72-h experimental period. Compared to the FW control group, 20‰ BW exposure significantly stimulated expressions of both *olgls1a* (77%) and *olgls2* (465%) after 72 h treatment (Fig. 4C,E). Nevertheless, 20‰ BW did not induce *olgls1b* expression at any of the treatment times (Fig. 4D). Similarly, glutamate-ammonia ligase (GLUL) enzymes are responsible for catalyzing glutamine synthesis (Fig. 4A). After transferring fish to 20‰ BW for 6 and 72 h, transcript expressions of *glul* were significantly upregulated compared to FW controls (Fig. 4F).

**Figure 5:**
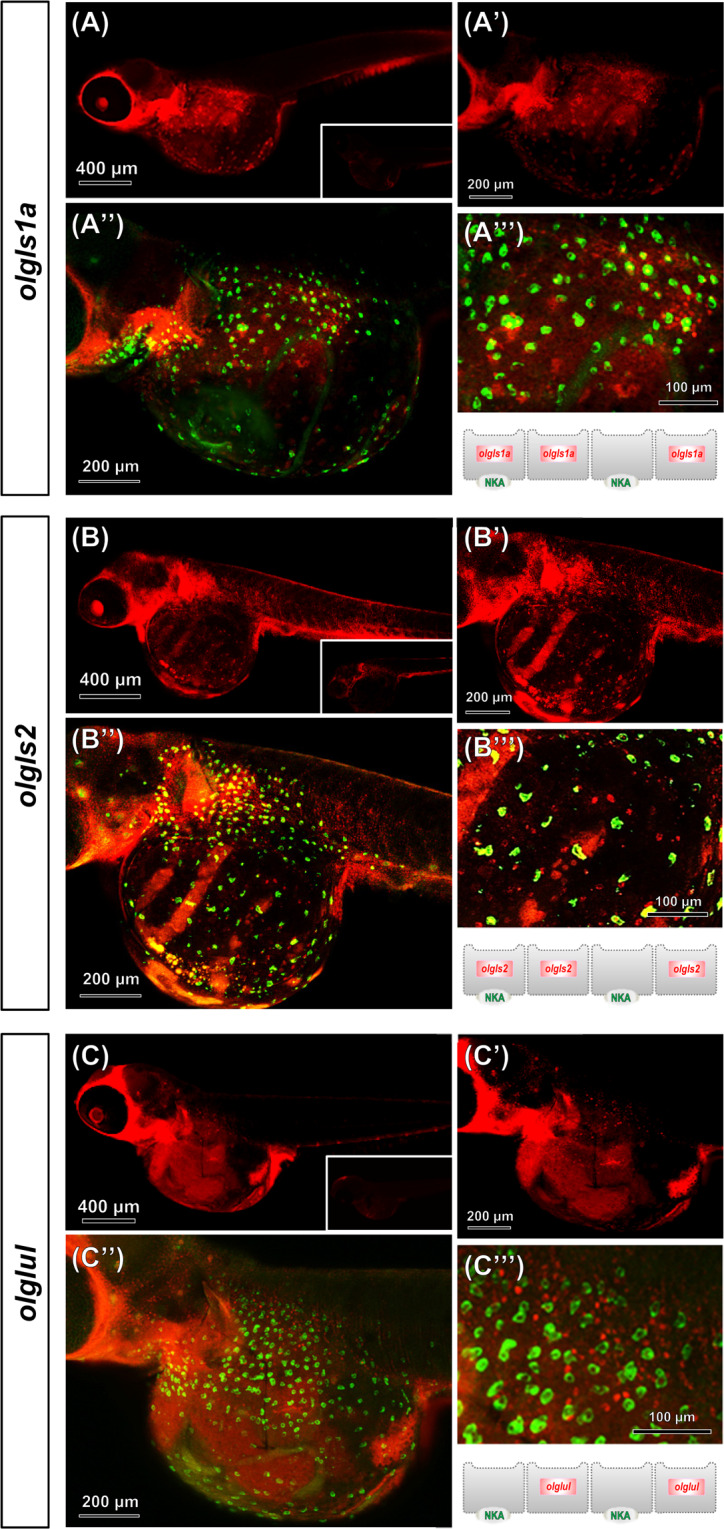
*In situ* hybridization and immunofluorescence double labeling of glutaminase (*olgls*) and glutamine synthetase mRNA with Na^+^/ K^+^-ATPase in yolk-sac epithelium of 7 dpf medaka larvae. Medaka larvae were double-labeled with mRNA antisense probes of *olgls1a* (**A** and A’), *olgls2* (**B** and B’), or *olglul* (C and C’) and Na^+^, K^+^-ATPase (NKA) α5 monoclonal antibody (merged image is shown in A”-C” and A”’-C”’). An illustration panel of the glutamate and glutamine transporters is represented below A”’-C”’. The inlay figures in (**A**–**C**) are sense probe hybridized images.

We further investigated the spatial expression of *olgls1a*, *olgls2*, and *olglul* in the epithelium of 7 dpf larvae by *in situ* hybridization. All three candidate genes showed the salt-and-pepper-like pattern that is typical of ionocytes in the yolk sac epithelium (Fig. 5A–C). On the one hand, *olgls1a* (Fig. 5A’ and A”) and *olgls2* (Fig. 5B’ and B”’) mRNA-positive cells were found to be 51% and 17% partially colocalized with the NKA-positive cells, respectively. On the other hand, mRNA expression of *olglul* (Fig. 5C’ and C”’) was not observed in NKA-positive cells, but localized in the neighboring epithelial cells.

### Expression of glutamate and glutamine transporters in fish gill epithelium after salinity challenge

**Figure 6:**
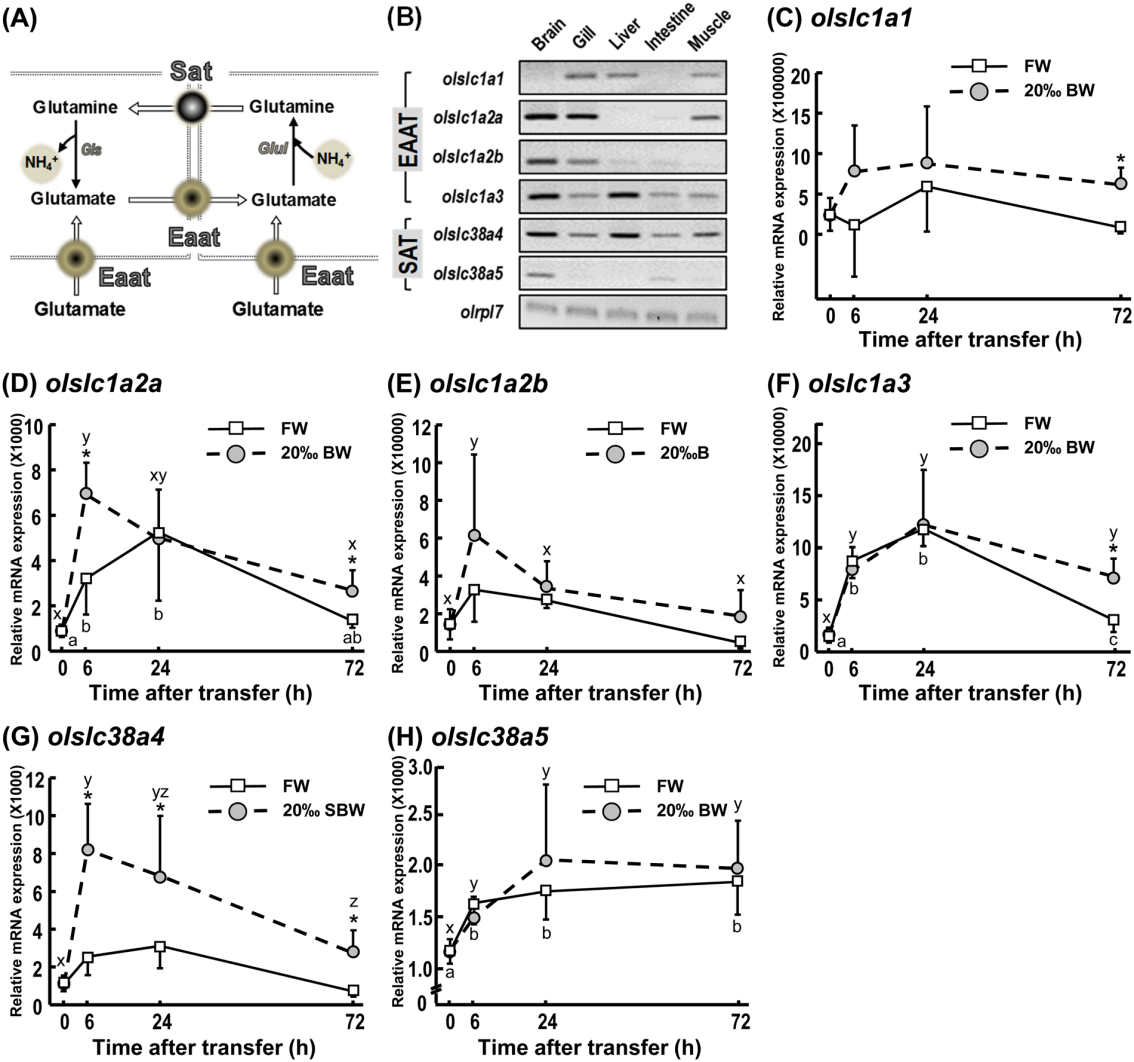
Gene expression of glutamate and glutamine transporters in gills during FW and 20‰ BW exposures. A schematic model is shown of the glutamate-glutamine cycle and putative transporters. (**A**) Cropped agarose gels (original images as shown in Supplemental Fig. S6) show semi-quantitative PCR (with 40-cycle amplification) of glutamate transporters (*olslc1a1*, *olslc1a2a*, *olslc1a2b* and *olslc1a3*), glutamine transporters (*olslc38a4* and *olslc38a5a*) and the reference gene ribosomal protein L7 (*olrpl7*) in brain, gill, liver, intestine and muscle of adult medaka. (**B**) Quantitative PCR (qPCR) analysis of relative mRNA expression levels of glutamate and glutamine transporters, *olslc1a1* (**C**), *olslc1a2a* (**D**), *olslc1a2b* (**E**), *olslc1a3* (**F**), *olslc38a4* (**G**), *olslc38a5* (H), in gills of adult medaka. *olrpl7* was used as the reference gene. Data are expressed as mean ± SD (n = 4–6). An asterisk (*) indicates significant difference, *p* < 0.05, between FW and 20‰ BW groups at the same time point. Different letters indicate significant differences between time points in each treatment group (Two-way ANOVA and Tukey’s HSD test). Overlapping letters indicate that differences are not significant between time points in the treatment group.

**Figure 7:**
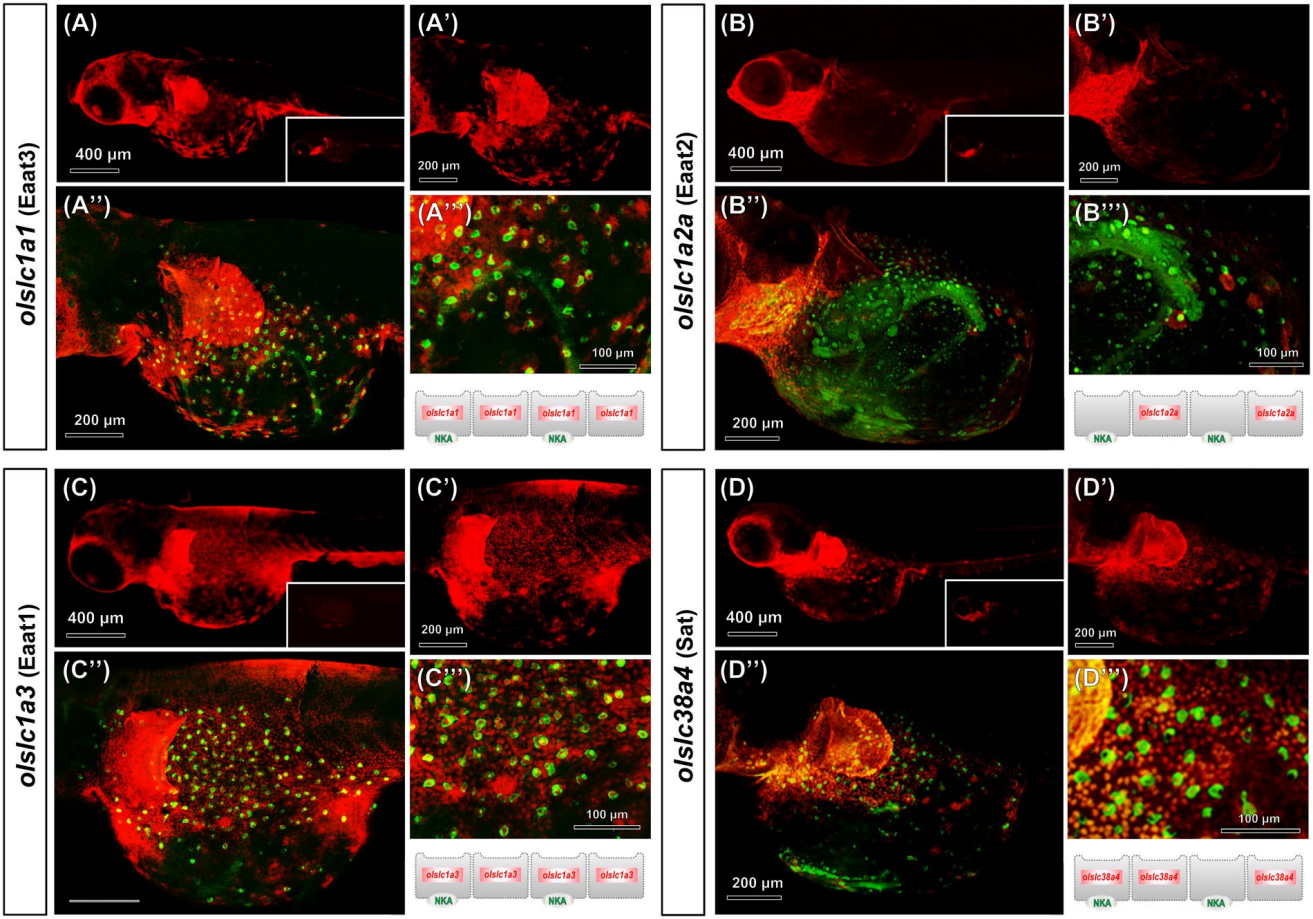
*In situ* hybridization and immunofluorescence double labeling of glutamate and glutamine transporters mRNA with Na^+^/ K^+^-ATPase in yolk sac epithelium of 7 dpf medaka larvae. Medaka larvae were double labeled with mRNA antisense probes of *olslc1a1* (**A** and A’), *olslc1a2a* (**B** and B’), *olslc1a3* (**C** and C’) or *olslc38a4* (**D** and D’) and Na^+^, K^+^-ATPase (NKA) α5 monoclonal antibody (merged image is shown in A”-D” and A”’-C”’). An illustration panel of the glutamate and glutamine transporters is represented below A”’-C”’. The inlay figures in (**A**–**D**) are sense probe hybridized images.

In this study, glutamate transporters (EAATs, *slc1a*) and glutamine transporters (SATs, *slc38a*) (Fig. 6A) were identified *in silico* via prediction with the ENSEMBL genome browser and cloned from medaka. Glutamate transporters (Eaats), *olslc1a1*, *olslc1a2a*, *olslc1a2b* and *olslc1a3*, and glutamine transporters (Sats), *olslc38a4* and *olslc38a5*, were all found to be expressed in medaka gills (Fig. 6B). Expressions of *olslc1a2a* and *olslc38a4* were comparatively higher in gill tissues than other Eaat and Sat paralogs (Supplemental Fig. S4C). Expression levels of glutamate transporters, *olslc1a1*, *olslc1a2a* and *olslc1a3*, were respectively increased by 151%, 71% and 92% after long-term (72 h) exposure to 20‰ BW (Fig. 6C,D.F), and *olslc1a2a*, was upregulated by about 122% 6 h after fish were transferred to 20‰ BW (Fig. 6D). In addition, the glutamine transporter, *olslc38a4*, was significantly upregulated in the 20‰ BW treatment group at all time-points examined (Fig. 6G). In another experiment, specific RNA probes were used for *in situ* hybridization to detect transcripts encoding glutamate/glutamine transport proteins, in addition to immunostaining for the epithelium ionocyte marker, NKA. *olslc1a1*, *olslc1a2b*, o*lslc1a3*, and *olslc38a4* were expressed in epidermal cells of the yolk sac and showed a typical ‘salt-and-pepper-like’ pattern of epidermal ionocyte staining (Fig. 7A–D). The glutamate transporter ortholog, Eaat3 (encoded by *olslc1a1*; Fig. 7A”and A”’), was partially (~61%) co-localized with NKA signals. Besides, all the Eaat1 (encoded by *olslc1a3*; Fig. 7C”and C”’) mRNA signals were found to be co-localized with NKA-positive cells and the neighboring epithelial cells in yolk sac epithelium. However, none of the *olslc1a2a*-expressing cells were also positive for NKA (Fig. 7B”and B”’). In addition, mRNA expression of the glutamine transporter, Sat (encoded by *olslc38a4*), was about 39% colocalized with NKA-labeled ionocytes, but this transporter was also expressed in neighboring epithelial cells (Fig. 7D”and D”’).

### Effects of SAT knockdown on Na^+^ flux from the epithelium

**Figure 8:**
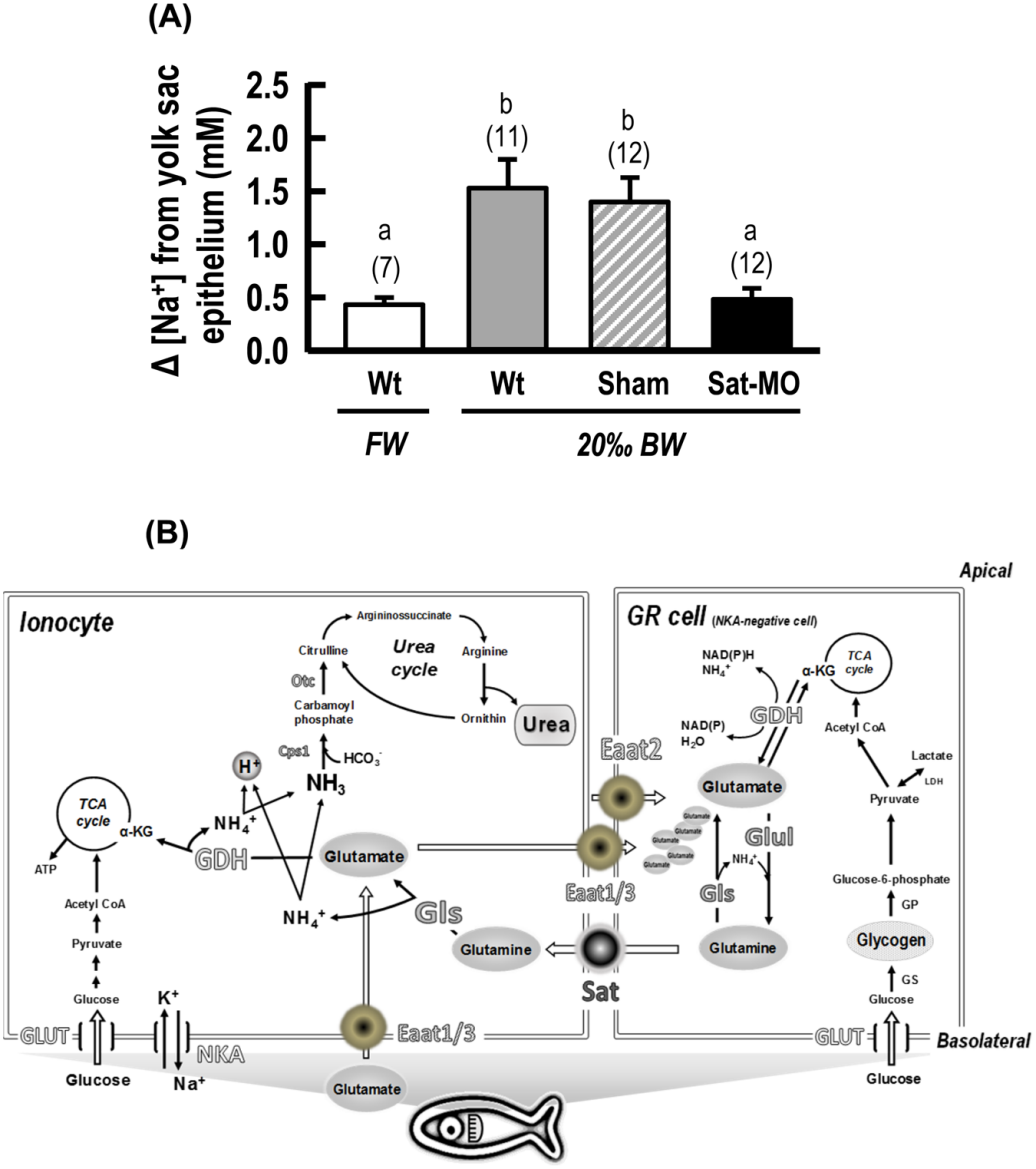
Abrogation of SAT on Na^+^ flux from the epithelium of 6 dpf medaka larvae and a schematic model of the proposed glutamate-glutamine cycle in epithelium of euryhaline teleosts. (**A**), Effects of SAT morpholino-modified antisense oligonucleotides (MOs) on Na^+^ flux from the 6 dpf medaka larvae yolk sac epithelium. Values are shown as mean ± SD. Different letters indicate significant differences among treatment groups (one-way ANOVA, Tukey’s pairwise comparisons). (**B**), Glutamate, a major plasma-derived energetic substrate, is transported to gills in response to salinity challenge. After euryhaline teleosts are acutely exposed to hyperosmotic BW, glutamate and glutamine are accumulated in gills via transport by glutamate transporter (Eaats) and glutamine transporter (Sat). Glutamine synthetase (Glul) is activated in energy storage GR cells to convert the glutamate to glutamine. Both glutaminase (Gls) and GLUL are activated, and using EAATs and SAT, glutamate/glutamine cycling occurs between epithelial ionocytes and neighboring GR cells. Intermediary production of NH_4_^+^ by glutamate/glutamine metabolism, involving carbamoyl phosphate synthetase (Cps1) and ornithine transcarbamylase (Otc), synthesizes urea to help maintain osmotic balance. Abbreviations: Eaat, excitatory amino acid transporter; Gls, glutaminase; GDH, glutamate dehydrogenase; Glul, glutamine synthetase; Glut, glucose transporter; GP, glycogen phosphorylase; GS, glycogen synthase; LDH, lactate dehydrogenase; αKG, α-ketoglutarate; NKA, Na^+^/K^+^-ATPase; Sat, sodium-coupled amino acid transporter; Cps, carbamoyl phosphate synthetases; Otc ornithine transcarbamylase; Ass, argininosuccinate synthetase; Asl, argininosuccinate lyase; Arg, arginase; NaR cell, Na^+^/ K^+^-ATPase-rich cell; GR cell, glycogen-rich cell.

Synthetic MO targeting Sat (encoded by *olslc38a4*) was injected into fertilized eggs to knockdown the translation of Sat protein (Supplemental Fig. S5A). The mortality rate of 1 ng Sat MO injected larvae was less than 20%, and the surviving morphants did not show significant abnormalities compared with wild-type (Wt) and sham control groups (Supplemental Fig. S5B). In FW, injection of 1 ng Sat MO did not produce significant changes in Na^+^ flux from epithelium in morphants compared with Wt and sham counterparts (Supplemental Fig. S5C). However, in the 20‰ BW environment, injection with 1 ng SAT MO significantly decreased Na^+^ flux (67%) from 6 dpf morphant epithelium compared to Wt and sham controls (Fig. 8A).

## Discussion

Glutamate is a central molecule in neurotransmission and brain metabolism^42,43^. It is not only the major excitatory neurotransmitter in the brain, but also serves roles as an energy substrate and protein constituent^44,45^. Here we elucidate the role of a glutamate-glutamine cycle in the branchial epithelium of teleosts that plays an important role the acclimation capacities to osmotic fluctuations.

### Osmoregulation in teleosts

It has been well documented that in euryhaline teleosts, acclimation to hyperosmotic SW requires timely activation of ion excretion and water retention mechanisms to maintain osmotic balance. Currently, it is thought that extra-renal organ functions are necessary for euryhaline teleosts to retain a relatively low osmotic concentration of body fluid under hyperosmotic conditions, such as in a marine environment^2,46–49^. It is generally accepted that in hyperosmotic SW conditions, gills actively secrete Na^+^, Cl^-^ and other ions into the extracellular space in order to maintain epithelial homeostasis^50,51^. Therefore, basolateral NKA in the epithelium may be highly important in providing the necessary driving force for ion transport^2^. In this study, we utilized medaka as a euryhaline model species and found that whole animal oxygen consumption rates were upregulated after 72 h 20‰ BW exposure. The increased oxygen consumption may reflect increased energetic demands for the transport of inorganic ions for the maintenance of osmotic homeostasis and was accompanied by an accumulation and retention of organic nitrogenous compounds, such as urea and trimethylamine oxide (TMAO). In elasmobranch fishes that utilize urea as an osmolyte, urea concentrations in tissues ranges from 200–400 µmol/g^52–54^. However, gill urea contents determined in the present study was approximately 0.7 µmol/g in 20% brackish water-treated fish suggesting that this increase in tissue urea concentration only has a very minor contribution as an osmolyte in this teleost species. Therefore, it is likely that an enhanced metabolism of nitrogenous organic compounds is primarily employed to fuel osmotic regulation.

In teleosts, ammonia can be excreted directly into the surrounding water, mostly through adult gills or larval skin^55^, and this ammonia excretion from the gill/skin epithelium is essential for nitrogen elimination. In our evaluation of medaka under hyperosmotic challenge, excretion of the potentially toxic NH_3_/NH_4_^+^ into the incubation water was not greatly increased; nevertheless, the NH_3_/NH_4_^+^ and harmless urea contents in gills were clearly increased. Intact metabolic O:N ratio was also significantly increased after 72 h of exposure to 20‰ BW, inferring that oxidative processes were elevated in comparison to those driving glutamine deamination to glutamate. Hence, the role of organic nitrogen metabolism in epithelial cells may be critical for the energetic requirements during acclimation to hyperosmotic conditions.

### Glutamate and glutamine metabolism in gills of euryhaline teleosts

Several environmental factors, including salinity and temperature, may affect AA regulation in various fish organs^5,56^. The rapid accumulation of AAs in fish gill suggests that they are transported to the tissue at a rate greater than the rate of utilization for energy production and protein synthesis. When AA concentrations in tissue increase, the rate of AA deamination increases as a result. Based on this study, not all the AA contents in gills were found to be responsive to hyperosmotic challenges (Supplemental Fig. S3). Accumulation of glutamate, glutamine and proline was observed in medaka gills after exposure to increased salinity, indicating that these amino acids are available as metabolic substrates for physiological processes under hyperosmotic challenge. In addition, glutamate, glutamine and proline are members of the glutamate family^57,58^, which infers that these AAs are easily trans-aminated into glutamate, and glutamate trans-deamination is the main pathway of AA oxidation. Earlier studies reported that GDH activity and glutamate content were increased in isolated gill epithelial cells of tilapia (*Oreochromis mossambicus*) following long-term SW acclimation^17^. Significant upregulation of key genes from the glutamate/glutamine transport pathways, including Eaat and Sat protein families, suggests that coincident effects on glutamate/glutamine metabolic pathways in medaka gills are activated when the fish are exposed to elevated environmental salinity. Here it should be noted that expression levels of some of the genes examined also increased in the control (freshwater) group along the experimental period of 72 h, suggesting a response of these genes to malnutrition as well. Bedsides, expression profiles of Eaats and Sats also infer that NKA-labeled epithelial ionocytes may take up extracellular glutamate and glutamine via these specific transporters. To generate a functional link between glutamate/glutamine transport and osmoregulation, Na^+^ secretion rates across the larval epithelium were measured in control animals and Sat morphants exposed to hyperosmotic conditions. These results indicate that a knock-down of Sat impairs Na^+^ secretion during exposure to increased environmental salinity providing direct evidence for the orchestrated action of nitrogenous energy metabolism and osmoregulation.

Regarding the catabolism of glutamate family AAs in fish, a series of studies have clearly demonstrated that the related metabolic machinery is not only used to generate α-ketoglutaric acid (α-KG) for TCA cycle, but that it is also important for the generation of ammonia^59^. The enzyme, Gls, converts glutamine into glutamate, generating ammonia for urea synthesis in mammals’ tissues. The expression patterns of glutamate and glutamine-converting genes, Gls and Glul, were stimulated by BW challenge in gill tissue in a time-dependent manner. In medaka hatchlings, there are at least two Gls or Glul isoforms expressed in ionocytes or neighboring cells, as shown by NKA-labeling experiments. Based on the spatial distribution of Gls and Glul in the epithelium of fish yolk sac, we infer that these enzymes may possibly be localized in the energy-storing GR cells of the epithelium, which were proposed to exist in tilapia and zebrafish^2,4,23^. As a consequence, it can be hypothesized that diverse isoforms of glutamate/glutamine-regulating enzymes exhibit different functions in teleost epithelium, and these isoforms differentially respond to hyperosmotic changes. Here it should be noted that despite the very similar overall function and cellular equipment of gill epithelia and the yolk integument^60–63^ we cannot rule out the possibility that there are some differences in gene expression patterns between larval and adult branchial epithelia.

Since GDH can bi-directionally catalyze glutamate degradation via deamination and glutamate formation via amination of α-KG with ammonia as a nitrogen source^64^, ammonia and glutamate contents are often closely correlated. Upon cellular metabolic induction, nitrogenous waste increases in parallel. However, these reactions are not usually considered to occur to a large extent in gill tissue. Instead, liver was postulated to the major organ for intact ammonia formation and exhibits relatively high GDH activity^59,65^. On the other hand, several studies have demonstrated that the excretion of ammonium/ammonia and production of urea could also be observed in other organs, such as kidney, intestine, muscle and brain^66–69^. Moreover, glutamate catabolism-related enzymes and specialized proteins to facilitate urea movement across the epithelium were also identified in fish gills^67–69^. Hence the activation of glutamate family AAs would provide necessary substrates for energy supply, osmotic balance and acid-base regulation^14,66,69–71^. In response to environmental osmolality elevation, significant accumulation of NH_3_/NH_4_^+^ and urea cycle-related enzymes (Cps and Otc) were observed in medaka gills; on the contrary, NH_3_/NH_4_^+^ excretion from whole fish was markedly decreased. These observations infer that the NH_3_/NH_4_^+^, which was released by glutamine deamination, was probably retained inside the epithelial cells. Reductions in NH_3_/NH_4_^+^ secretion are a reasonable response, given the necessity to secrete sodium and chloride under hyperosmotic conditions. In the yolk epithelium of euryhaline medaka it has been suggested that ammonia excretion is mediated by rhesus proteins coupled to Na^+^/H^+^ exchange activity in the apical membrane^41,60^. Thus, enhanced ammonium excretion would be counterproductive due to an uptake of Na^+^ by this process and activation of the urea cycle may help to detoxify the NH_3_/NH_4_^+^ by the formation of urea. Here our results provide first in depth knowledge for the molecular basis of glutamate/glutamine transporters, metabolic enzymes and nitrogenous waste products, in ion regulatory epithelia of euryhaline fish with relevance for salinity acclimation capacities.

## Conclusion

The present work demonstrated that euryhaline teleosts have evolved an efficient glutamate/glutamine metabolic machinery in their branchial epithelium to maintain osmoregulation in the face of environmental osmolality disturbances (Fig. 8B). This study is the first to elucidate how the non-essential AAs, glutamate and glutamine, are potentially involved in NH_3_/NH_4_^+^ production and urea accumulation in gills, providing novel insights into the energetics of osmoregulation. Furthermore, the expression patterns of glutamate/glutamine transporters and catabolism regulators, Eaat, Sat, Gls and Glul, in the epithelium of medaka larvae not only offer novel insights into potential AA-transport routes between functionally distinct epithelial cells, but also begins to elucidate the molecular and cellular utilization of glutamate/glutamine in fish osmoregulation. Moreover, the present study suggests that features of a glutamate-glutamine cycle may be commonly derived from epidermal development, since they are found in both neural ectoderm-derived CNS and the non-neural ectoderm-derived gill epithelium in vertebrates. These findings highlight the importance of NH_4_^+^-based urea production via glutamate/glutamine metabolism that contributes to the energetics of well-developed osmoregulatory abilities in euryhaline teleosts.

## Supplementary information

Supplementary Information.
