# Supplementary material for: Energy and nitrogenous waste from glutamate/glutamine catabolism facilitates acute osmotic adjustment in non-neuroectodermal branchial cells
Source: Sci Rep. 2020 Jun 11;10:9460. doi: 10.1038/s41598-020-65913-1 (PMC7289822; doi:10.1038/s41598-020-65913-1)
Supplement: Supplementary file 1 — Supplementary Information. [file 41598_2020_65913_MOESM1_ESM.pdf]

Supplemental information to:

Energy and nitrogenous waste from glutamate/glutamine catabolism facilitates acute osmotic adjustment in non-neuroectodermal branchial cells

Pei-Chen Huang[1], Tzu-Yen Liu[1], Marian Y. Hu[2], Isabel Casties[3], and Yung-Che Tseng[1#]

1. Marine Research Station, Institute of Cellular and organismic Biology, Academia Sinica, I-Lan County, Taiwan, Republic of China (ROC)
2. Institute of Physiology, Christian-Albrechts University Kiel, Kiel, Germany
3. Helmholtz Centre for Ocean Research Kiel (GEOMAR), Kiel, Germany

*To whom correspondence should be addressed:

E-Mail: yctseng@gate.sinica.edu.tw

Tel: +886-3-9880544#14

Supplemental Figure S1

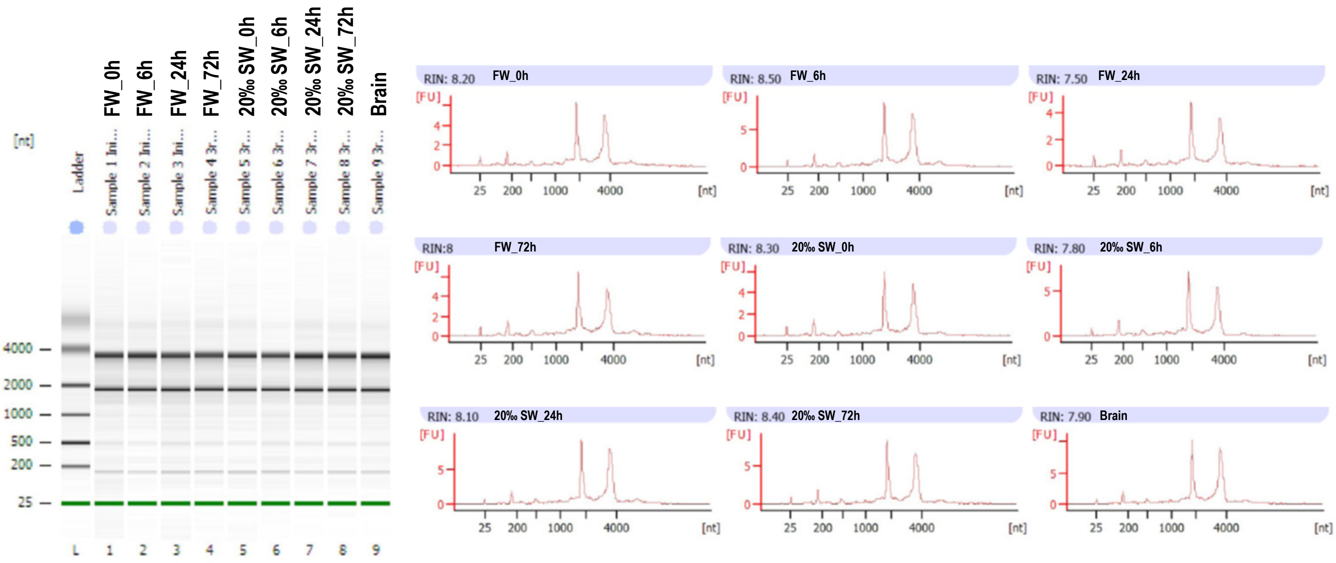

Supplemental Figure S1. Bioanalyzer electropherograms of examined RNA samples.
Randomly selected one of gill samples respectively collected from the FW- and 20‰ SW time course-treated medaka and applied on the Agilent 2100 bioanalyzer (Agilent Technologies, Santa Clara, CA) to stringent estimate the RNA quality. Brain tissue for tissue-scan analysis was also selected as a reference sample. The Agilent 2100 Bioanalyzer generates an electropherogram, and displays results of the RNA Integrity Number (RIN). The RIN software permits the classification of total RNA, based on a numbering system from 1 to 10, with 1 being the most degraded profile and 10 being the most intact.

Supplemental Figure S2

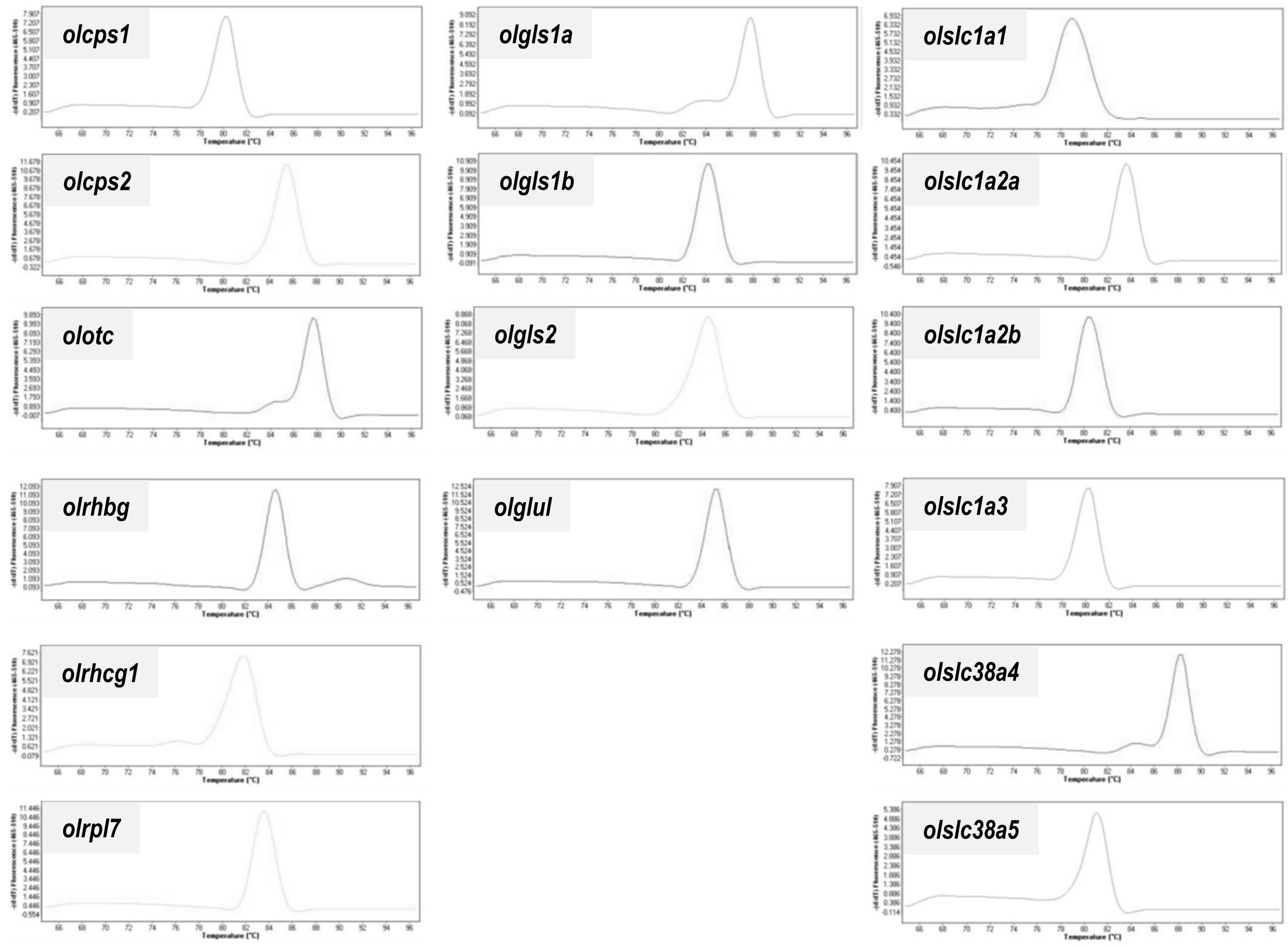

Supplemental Figure S2. Melting curve of candidate genes under qPCR analysis in gills of adult medaka.

Each melting curve of estimated genes as listed in Supplemental Table S1 in gills of adult medaka was acquired from qPCR analysis.

Supplemental Figure S3

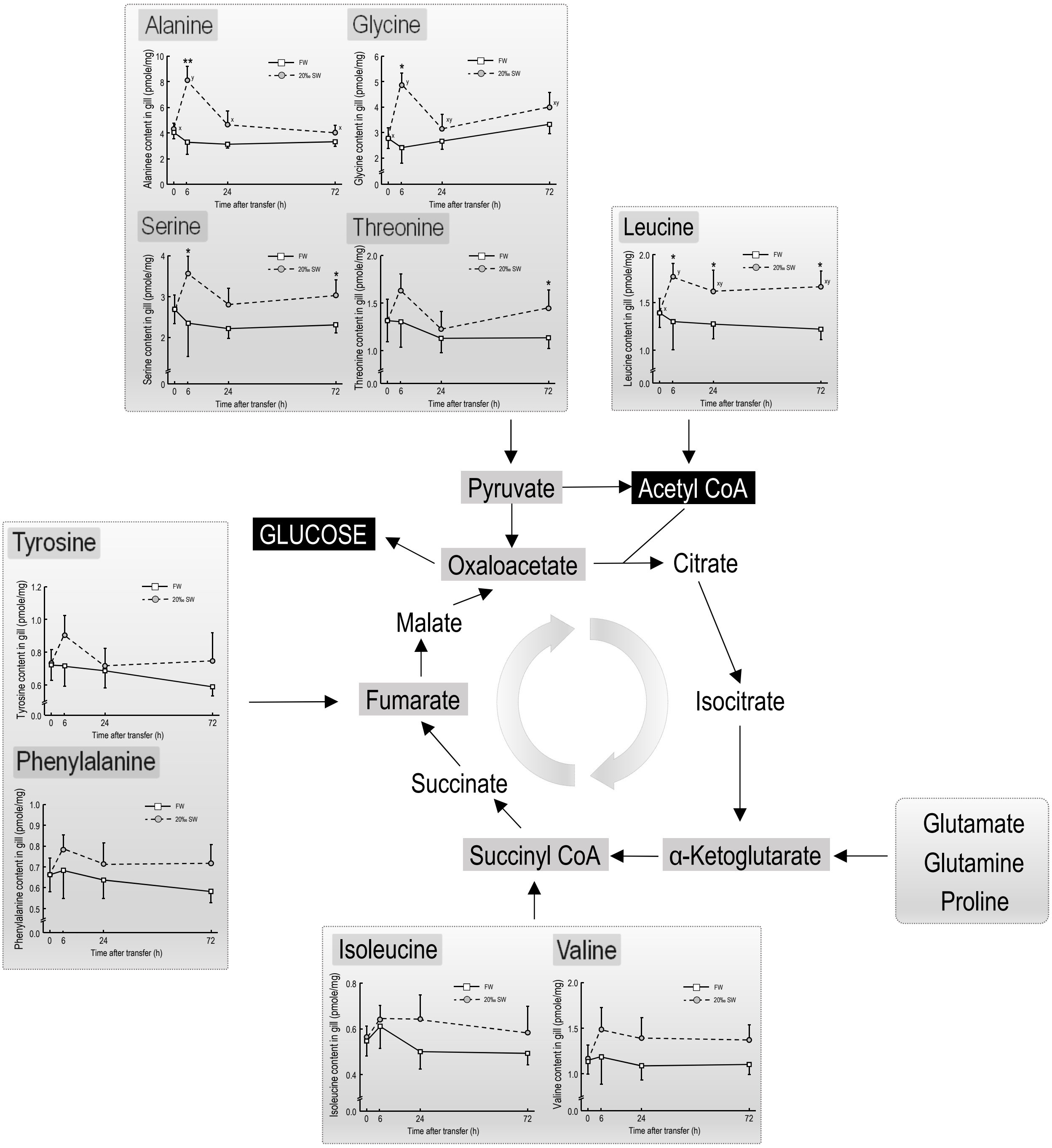

Supplemental Figure S3. Carbon cycle related amino acids (pmole/μg) contents in gills during FW and 20 ‰ SW exposures.
Contents of alanine, glycine, serine, threonine (metabolized to pyruvate), leucine (metabolized to acetyl CoA), tyrosine, phenylalanine (metabolized to fumarate), isoleucine, valine phenylalanine (metabolized to succinyl CoA) in the gills of medaka were measured by ultra-performance liquid chromatography (UPLC). Data are expressed as mean ± SD (n = 4-6). Asterisk indicates significant difference (*$p < 0.05$, **$p < 0.01$) at same time point between FW and 20 ‰ SW groups. Different letters indicate significant differences between time points in each treatment group (one-way ANOVA, Tukey's pairwise comparisons).

Supplemental Figure S4

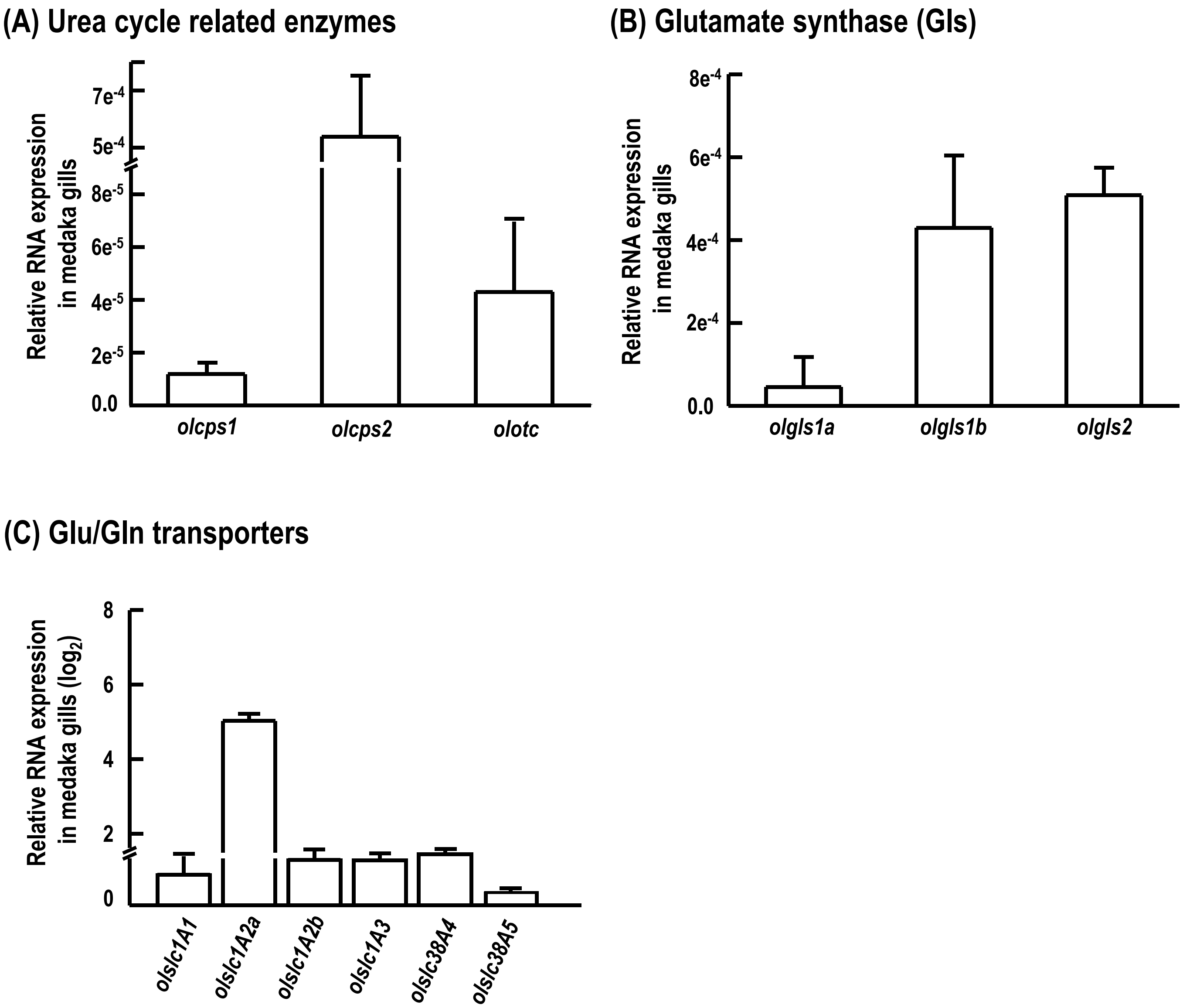

Supplemental Figure S4. Related transcript expressions of glutamate/glutamine related transporters/enzymes and urea cycle related enzymes in medaka gills.
Qualitative PCR (qPCR) analysis of relative mRNA expression levels of urea cycle related enzymes (A), glutaminase (Gls, B), and glutamate and glutamine transporters (C) in gills of adult medaka. Data are expressed as means ± SD (n=4-6).

Supplemental Figure S5

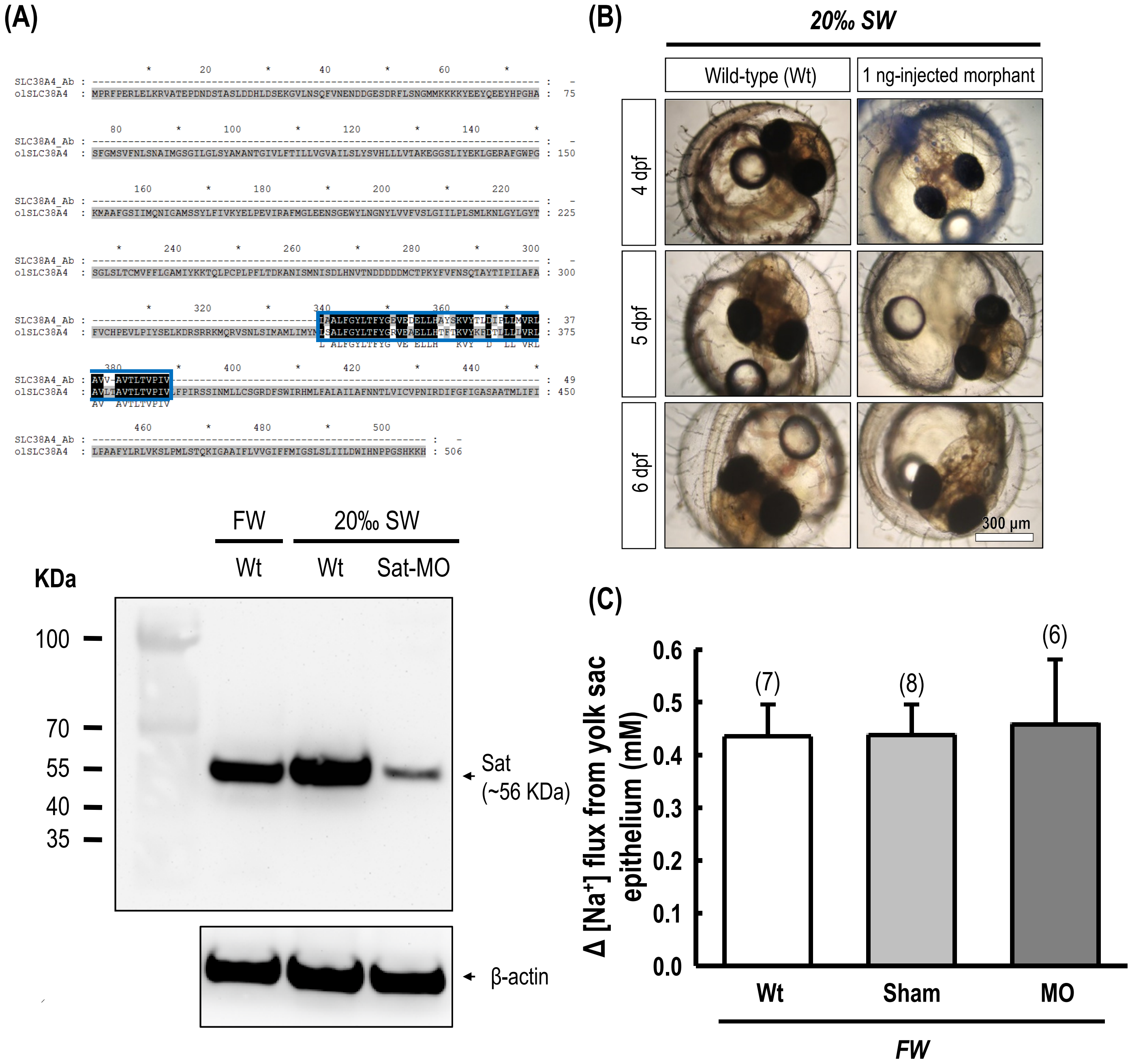

Supplemental Figure S5. Effects of abolishment of *olslc38a4* (SAT) on phenotypes, Na^+ flux from epithelium , and protein expression in medaka larvae.
(A), Deduced sequence alignment of medaka Sat with the commercial anti-SLC38A4 antibody immunogen peptide (a synthetic peptide directed towards the human SLC38A4; Sequence homology: 74%; ab58785; Abcam Cambridge, UK). Western blot analysis of the SAT-abolished effects on protein expression in Sat morphants. 6 dpf medaka larvae homogenates (collected from the wild-type in FW, wild-type in 20‰ SW, and Sat morphants in 20‰ SW) were respectively applied and indicate the commercial anti-SLC38A4 antibody could detect proteins from different samples of medaka larvae in the expected molecular weight size about 56 KDa. (B), Light microscopic images of wild-type (Wt) and 1 ng SAT MO injected medaka embryos under 20‰ SW condition. (C), Effects of SAT MO injection on Na^+ flux compared with that in wild-type and sham control ones in 6 dpf medaka larvae under freshwater (FW) environment. Values are presented as means ± SD and were compared using Student's t-test. A significant difference was accepted at $p < 0.05$.

Supplemental Figure S6

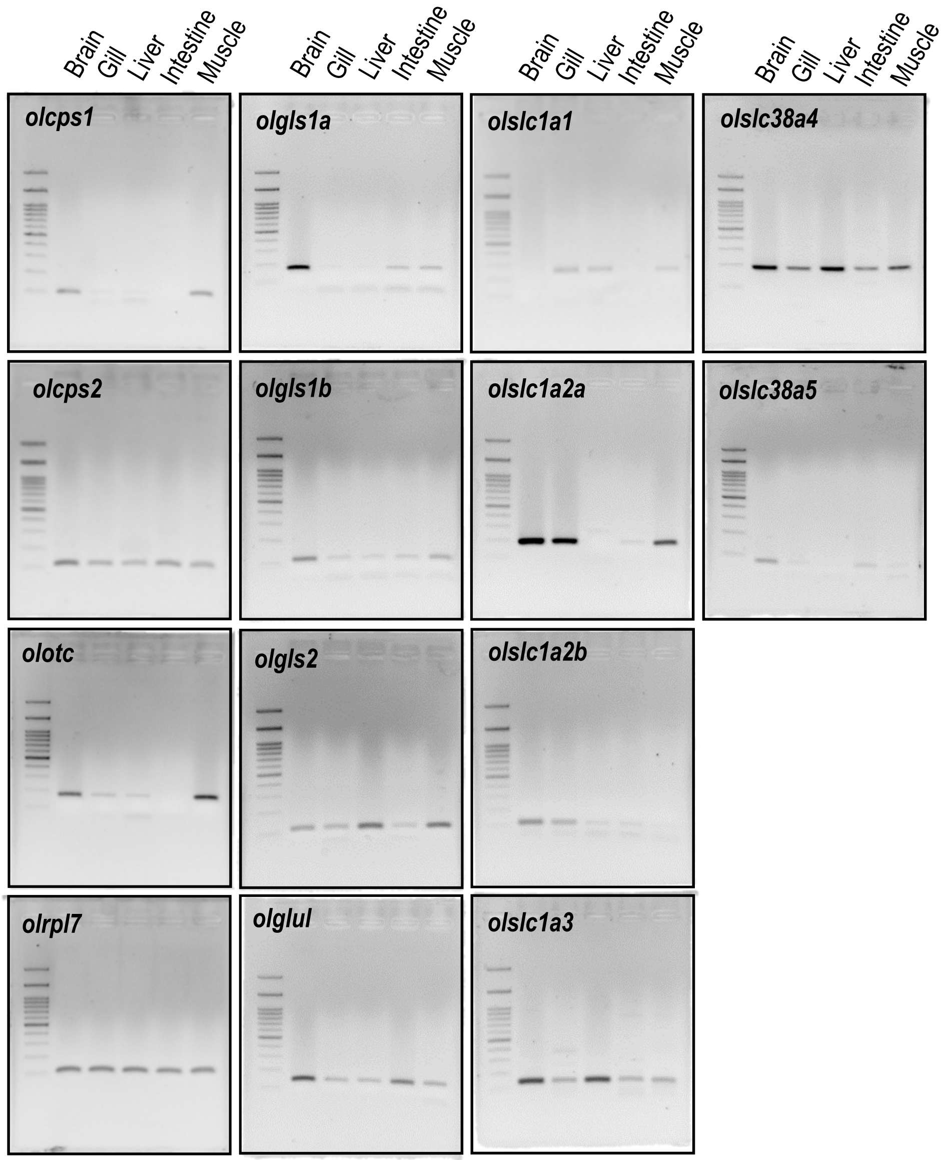

Supplemental Figure S6. Transcript expression patterns among different tissues of adult medaka. Full-length agarose gels show semi-quantitative PCR (with 40-cycle amplification) of urea cycle related enzymes, glutamate/glutamine synthesis/transport related candidates, and the reference gene ribosomal protein L7 (*olrpl7*) in brain, gill, liver, intestine and muscle of adult medaka.

Supplemental Table 1 Primers for RNA *in situ* hybridization probe synthesis

Protein name		Gene name		Primer sequence (5'->3')	Amplicon size (bp)
**Glutamate/ Glutamine cycle**	Glutamate transporter	*olslc1a1*	F	GTTCTTGGAGATGCTTAC	625
			R	CAGTAGGGTTAGGGTTTA	
		olslc1a2a	F	TGGACGGAACTGCTCTGTATGA	509
			R	GAAGATCTCTGCGCTGCCATTATT	
		olslc1a3	F	GAGAAGGAGAGGAAGAAA	553
			R	CATGAGGACAGTGAAATG	
	Glutamine transporter	*olslc38a4*	F	GAGCGCCGGTGTTAAAGGATTA	645
			R	ACGCAGTGGAAATGCTGGATAGA	
	Glutamine synthetase	*olglul*	F	TATCTCCTTCCTGCGGCTATGT	551
			R	ATCTCCTTGGTGCTGACGTTTG	
	Glutaminase	*olgls1a*	F	TGCGTGAAGCCGCTGAAATA	562
			R	AAGCGAACTGTCCCGAGAAATC	
		olgls2	F	GTCGCTAAATGAAGAAGG	288
			R	GAGCACAACTGAAAGTAG	

F, forward primer; R, reverse primer
